# Immune checkpoint expression and relationships to anti-PD-L1 immune checkpoint blockade cancer immunotherapy efficacy in aged versus young mice

**DOI:** 10.1002/aac2.12045

**Published:** 2022-02-25

**Authors:** Myrna G. Garcia, Yilun Deng, Clare Murray, Ryan M Reyes, Alvaro Padron, Haiyan Bai, Aravind Kancharla, Harshita Gupta, Shai Shen-Orr, Tyler J. Curiel

**Affiliations:** ^1^South Texas Medical Scientist Training Program, University of Texas Health, San Antonio, Texas, USA; ^2^Graduate School of Biomedical Sciences, University of Texas Health, San Antonio, Texas, USA; ^3^Department of Medicine, University of Texas Health, San Antonio, Texas, USA; ^4^Senda Biosciences, Cambridge, MA, USA; ^5^Technion, Israel Institute of Technology, Haifa, Israel; ^6^Clayton Foundation for Research, Houston, Texas, USA; ^7^Mays Cancer Center, University of Texas Health, San Antonio, Texas, USA

**Keywords:** age, cancer immunotherapy, immune checkpoints, interferon-*γ*, PD-1, PD-L1

## Abstract

**Introduction:**

Aging is the biggest cancer risk, and immune checkpoint (IC) inhibition (ICI) is a revolutionary cancer immunotherapy approach. Nonetheless, there are limited preclinical/clinical data regarding aging effects on ICI outcomes or age effects on IC expression in different organs or tumors.

**Methods:**

Flow cytometry assessed IC on immune and non-immune cells in various organs in young and aged BL6 mice. Comparisons: aged versus young naïve WT versus interferon-*γ*^KO^ mice and WT challenged with B16F10 melanoma and treated with *α*PD-1 or *α*PD-L1 ICI. We co-cultured young and aged T cells and myeloid cells in vitro and used OMIQ analyses to test cell–cell interactions.

**Results:**

*α*PD-1 ICI treated melanoma in young and aged hosts, whereas *α*PD-L1 ICI was only effective in young. We found considerable, previously undescribed age effects on expression of various IC molecules participating in the ICI treatment, including PD-1, PD-L1, PD-L2, and CD80, in distinct organs and in the tumor. These data help explain differential ICI efficacy in young and aged hosts. Host interferon-*γ* influenced age effects on IC expression in both directions depending on specific IC molecule and tissue. IC expression was further affected by tumor challenge on immune, non-immune, and tumor cells in tumor and other organs. In in vitro co-culture, *α*PD-1 versus *α*PD-L1 distinctly influenced polyclonal T cells in young versus aged, suggesting mechanisms for distinct age-related ICI outcomes.

**Conclusion:**

Age affects IC expression on specific immune cells in an organ- and tissue-specific manner. ICs were generally higher on aged immune cells. High immune-cell PD-1 could help explain *α*PD-1 efficacy in aged. High co-expression of CD80 with PD-L1 on dendritic cells could help explain lack of *α*PD-L1 efficacy in aged hosts. Factors other than myeloid cells and interferon-*γ* also affect age-related IC expression and T cell function, meriting additional studies.

## 1 | BACKGROUND

As human lifespan has increased, there has also been a shift in the diseases that have the highest burden on industrial societies today.^1^ In the United States, for example, cancer is the leading cause of death for individuals 55–74 years of age and the second leading cause of death overall.^2,3^ Additionally, age is currently the biggest single risk factor for developing most cancer types, including melanoma.^4^

The immune system plays a major role in melanoma outcomes.^5,6^ For example, immune checkpoint inhibition (ICI) as cancer immunotherapy has shown remarkable efficacy in improving T cell responses and prolonging survival in melanoma patients.^7^ ICI currently consists of antibodies blocking the IC PD-1, PD-L1, or CTLA-4, all of which are approved to treat melanoma.

ICI cancer immunotherapy has revolutionized cancer treatment,^8^ with FDA approval of eight monoclonal antibodies to date blocking the PD-1, PD-L1, or CTLA-4 ICs with many more under investigation. While ICI is greatly successful at treating many cancer types, most patients do not respond, and response mechanisms and biomarkers are not well understood. Furthermore, pre-clinical studies testing the efficacy of ICI were initially done on young hosts and more recent studies, including our own, highlight differences in response to cancer immunotherapy in young versus aged,^9–13^ but more detailed studies are needed. Clinical data on ICI efficacy in elderly individuals with cancer are encouraging but inconclusive, with retrospective meta-analyses showing similar or improved efficacy in aged (> 60 years) versus young (< 60 years) melanoma patients treated with ICI agents,^14–18^ while another study showed no significant progression free survival (PFS) improvement in cancer patients ≥ 75 years of age treated with ICI versus control groups, while younger patients (< 75 years) had significant PFS improvement.^19^ Despite contradictory data, age-related differences likely occur, but clinical studies could be underpowered due to low enrollment of elderly adults into clinical trials^20^ or age effects on ICI in humans do not manifest until ages greater than those thus far studied.

Age brings significant changes in immunity, more than just declines in specific functions, but also encompasess acquisition of new functions, appearance of novel immune subsets, IC expression on cells not generally expressing them in the young, and changes in host non-immune compartments.^21^ These age-related changes in immunity likely contribute to ICI response in the aged.

We previously tested ICI outcomes in young versus aged mice and found that while *α*PD-1 ICI treats aged and young mice with B16F10 melanoma, *α*PD-L1 ICI only treats young with B16F10 melanoma.^10^ Until this finding, effects of *α*PD-1 and *α*PD-L1 ICI have been considered dichotomous: *α*PD-1 and *α*PD-L1 either both work or both fail as ICI for a given cancer. Our data suggest specific differences in young versus aged that could account for this dichotomy. As PD-1 is ligated by both PD-L1 and PD-L2,^22–24^ and PD-L1 engages PD-1 and CD80,^25^ age-related treatment dichotomies we discovered could be related to specific IC expression or signal differences blocked by these ICI treatments. We therefore investigated differential expression of PD-1, PD-L1, PD-L2, and CD80 ICs in young versus aged tumor-naïve and tumor-challenged mice. We tested ICI outcomes in a murine melanoma model (B16F10) that is difficult to treat with single-agent ICI immunotherapy.^26^

## 2 | METHODS

### 2.1 | Mice

Male and female wild-type (WT) C57BL/6J (BL6) mice were purchased from Jackson Laboratory (Bar Harbor, ME, USA). Genetic interferon-*γ* knock out mice on the BL6 background (B6.129S7-Ifng^tm1Ts^/J, [IFN*γ* ^KO^]) were purchased from Jackson. Mice were purchased at ages 6–8 weeks and aged at our institutional facility, maintained under specific pathogen-free conditions, and given food and water ad libitum throughout their lives. Age- and sex-matched mice that were young (2– 4 months) or aged (19–30 months) were used for all experiments.

### 2.2 | In vivo tumor challenges, treatments, and assessments

0.5 × 10^6^ B16F10 cells in 150 *μ*l sterile PBS were injected into sterilized mouse flanks intradermally with a 27-gauge needle. Intradermal tumor size was measured every other day using vernier calipers and volume calculated as (length × width^2^)/2.^27^ Treatments were *α*PD-L1 (10F.9G2) 100 *μ*g/mouse on days (after B16 challenge) 7, 11, and 15, *α*PD-1 (RMP1–14) 100 *μ*g/mouse on days 7, 11, 15 or 7, 10, 13 or isotype controls. Mice were sacrificed around day 15–17, when tumors reached > 1,500 mm^3^ for survival or ∼400 mm^3^ for immune studies.^10,12^ Distressed mice (ruffled fur, gait or posture disturbance, skin or tumor ulcers) were sacrificed irrespective of tumor sizes, which counted as day of death for survival studies.

### 2.3 | Flow cytometry

Mice were sacrificed by cervical dislocation after induction of deep isoflurane anesthesia. Tumors were dissected and placed in 100 *μ*m cell strainers and filtered into single-cell suspensions in serum-free RPMI-160 medium. For IC studies of tumor-naïve mice, young or aged mice were sacrificed and bone marrow (BM) cells were collected from femurs and tibias as previously published.^28^ Spleens were placed in 100 *μ*m cell strainers and filtered into single-cell suspensions in the serum-free RPMI-160 medium. Red blood cell lysis was performed by hypotonic shock. Briefly, splenocytes were placed in 900 *μ*l of deionized H_2_O for 30 s and then 100 *μ*l of 10× dPBS was added. Cells were counted with a Vi-cell XR (Beckman Coulter) and 3–5 × 10^6^ cells/sample were transferred to 96-well plates. Dead cells were excluded by LIVE/DEAD Fixable Blue Dead Cell Stain Kit for ultraviolet excitation (Thermo Fisher Scientific) or Ghost Dye Violet 510 (Tonbo Biosciences). Non-specific labeling was pre-blocked with anti-CD16/32 at 1:100 dilution (clone 93, Biolegend). Single-cell suspensions in 1× PBS supplemented with 2% FBS were stained with monoclonal antibodies. For tumor ICI studies, monoclonal flow cytometry antibodies used were *α*PD-L2 (APC, TY25), *α*CD8 (APC-CY7, 53–6.7), *α*CD45 (FITC, 30F11), *α*CD4 (BV605, GK1.5), *α*PD-1 (BV711, RMP1–30), *α*PD-L1 (PE-CY7, MIH7), and *α*CD3 (BUV 737, 17A2). For IC studies on tumor-naïve young and aged mice, monoclonal flow antibodies used were *α*CD45 (BUV 496, 30F11), *α*CD3 (BV 510, 17A2), *α*CD4 (AF532, GK1.5), *α*CD8 (BUV 395, 53–6.7), *α*CD11b (PERCP CY 5.5, M1/70), *α*CD11c (SB 645, N418), *α*F4/80 (BB700, T45–2342), *α*B220 (BUV 661, RA3–6B2), *α*PD-L1 (BV 421, MIH7), *α*PD-L2 (BV 605, TY25), *α*PD-1 (PE/DAZZLE 594, RMP1–30), *α*CD80 (BUV 563, 16–10A1).

For intracellular staining, cells were fixed and permeabilized with a FoxP3/transcription factor buffer kit (eBioscience, San Diego, CA) according to manufacturer instructions, and incubated at 4°C for 30–45 min. For cytokine detection, cells were stimulated with Cell Activator Cocktail (Biolegend) containing phorbol 12-myristate 13-acetate, ionomycin, and brefeldin at 2 *μ*l cock-tail/mL in CR10 medium (RPMI-1640 with 10% FBS, l-glutamine, sodium pyruvate, non-essential amino acids, penicillin/streptomycin, and HEPES buffer) for 6–8 h in a 37°C incubator. After stimulation and permeabilization, intracellular staining was performed by incubating cells at 4°C for 30–45 min with antibodies. Events were acquired using BD ARIA or Cytek Aurora hardware and analyzed by FlowJo (FlowJo LLC, Ashland, OR, USA) or OMIQ software.

### 2.4 | Ex vivo co-culture assay

Spleens were collected from naïve young and aged mice and placed in serum-free RPMI-1640 medium. Single-cell suspensions were generated by crushing tissue and filtering through a 100 *μ*m strainer. Red blood cells were lysed in 900 *μ*l water for 30 s followed by the addition of 100 *μ*l 10× PBS. Following red blood cell lysis, splenocytes were stained with monoclonal antibodies for CD4, CD8 and CD11b (clones and colors as below) and sorted using a BD ARIA sorter. CD4^+^ and CD8^+^ cells were stained with CellTrace CFSE (ThermoFisher) and resuspended in CR10 medium (RPMI-1640 with 10% FBS, 1% l-glutamine, sodium pyruvate, non-essential amino acids, penicillin/streptomycin, and HEPES buffer). Sorted cells were plated at a ratio of 100,000 CD3^+^:20,000 CD11b^+^ cells and stimulated with *α*CD3/CD28 Dynabeads (ThermoFisher; 1 bead/cell). Additionally, monoclonal antibodies: *α*PD-1 (RMP1–14), *α*PD-L1 (10F.9G2), *α*CD80 (16–10A1), or isotype controls were added to wells at a concentration of 20 ng/ml. After 72 h, cells were washed and labeled for flow cytometry. Flow cytometry monoclonal antibodies used were *α*CD4 (BUV 496, GK1.5), *α*IFN*γ* (BUV 737, XMG1.2), *α*PD-L1 (BV 421, MIH7), *α*B220 (BV 510, RA3–6B2), *α*PD-L2 (BV 605, TY25), *α*CD69 (BV 650, HI.2F3), *α*PD-1 (BV 711, RMP1–30), *α*CD3 (BV 786, 17A2), *α*CD11b (PERCP CY 5.5, M1/70), *α*IL-2 (PE, JES6–5H4), *α*CD11c (PE CY7, N418), *α*CD80 (APC, 16–10A1), *α*CD8 (AF 700, 53–6.7).

### 2.5 | Statistics and data analysis

We used an effect size of 0.85 to determine that the two-sample *t*-test achieves 80% power with 11 mice per B16 group to detect a survival difference between treatment and control with *α*PD-1 at 14 days with a two-sided *α* = 0.05. Statistical analyses were conducted with Prizm software (GraphPad). Data in immune analyses are means ± standard error of mean (SEM). For tumor growth, we used two-way ANOVA plus Bonferroni post-tests to compare replicate means. Heat map analysis was performed from flow cytometry data using OMIQ software. For Figure 9A, manually gated CD3^+^ T cell populations were used to generate a heatmap on the basis of T cell markers shown in the figure. Hierarchical clustering was used on heatmap to generate a row dendrogram. For all other analyses, one-way ANOVA or unpaired Student’s *t*-test was used as indicated in figure legends. *P* ≤ 0.05 was considered significant.

## 3 | RESULTS

### 3.1 | *α*PD-1 ICI treats B16F10 melanoma in young and aged mice while *α*PD-L1 ICI only treats young

**FIGURE 1:**
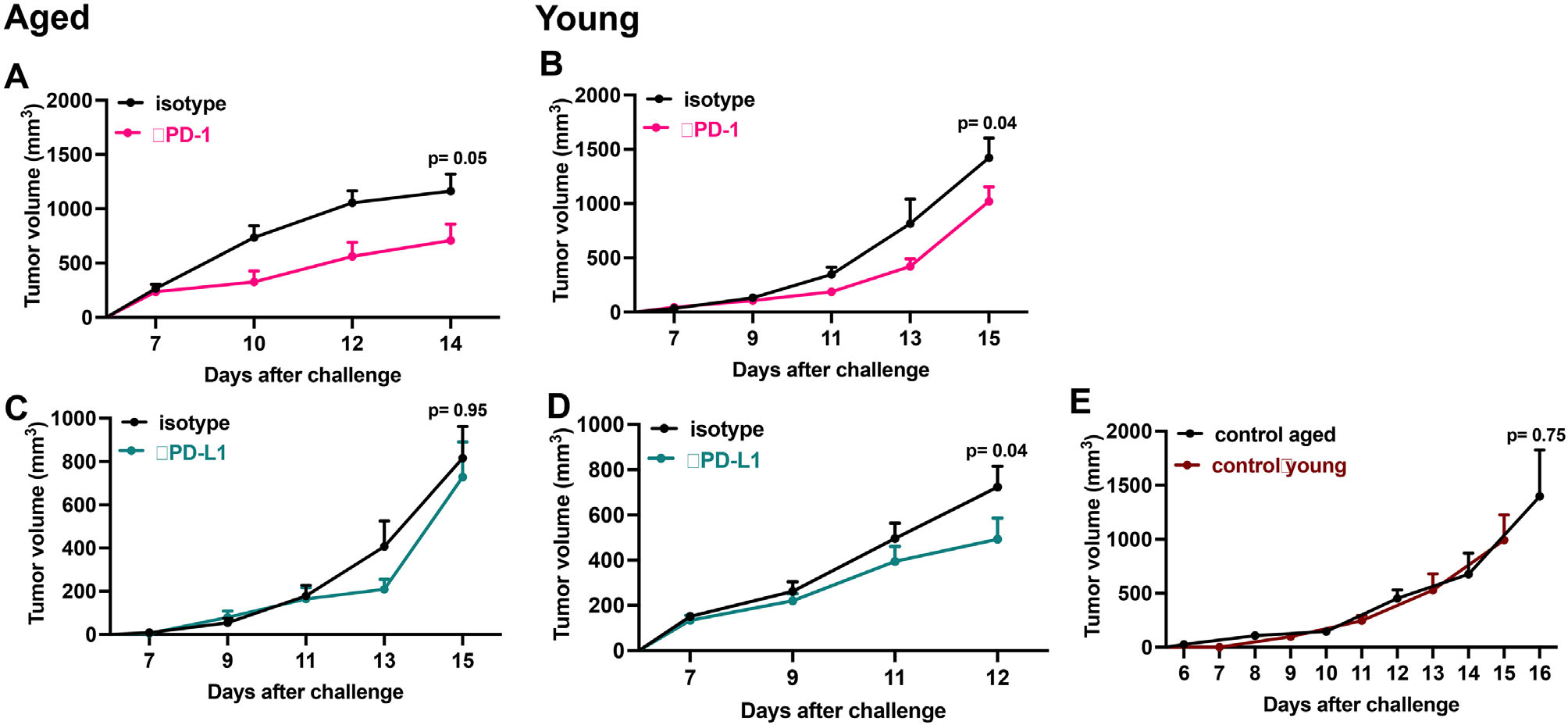
*α*PD-1 but not *α*PD-L1 treats aged mice challenged with B16 F10 melanoma cells. Tumor growth in (A, C) wild-type aged (24–26 months) male mice and (B, D) wild-type young male mice (2–4 months) challenged orthotopically with 0.5 × 10^6^ B16 F10 cells and treated with (A, B) *α*PD-1 (100 *μ*g/ mouse) (C,D) *α*PD-L1 (100 *μ*g/ mouse), (A–D, E control young) isotype control (100 *μ*g/ mouse) or (E control aged) treated with deoxidized phosphate buffered saline (150 *μ*l/mouse) intra-peritoneally every 4 days starting on day 7. Standard error of mean indicated; *P*-values, two-way ANOVA. *n* = 4–13 tumors/group

We confirmed that young and aged mice in these experimental cohorts challenged with B16F10 melanoma respond to *α*PD-1 ICI, whereas only young respond to *α*PD-L1 (Figure 1A–D) as we previously reported.^10^ Of note, tumor growth was not significantly different in isotype controls for *α*PD-1 (Figure 1A,B) or *α*PD-L1 (Figure 1C,D) and or control treated young versus aged mice (Figure 1E) demonstrating no significant age effect on tumor growth in these mice, contrasting with some reports.^29,30^

### 3.2 | IC expression in tumor-naïve aged mice differs by age, immune cell type, and tissue

Given the age-related treatment dichotomy observed using *α*PD-1 versus *α*PD-L1 ICI, we performed a survey of young versus aged, naïve mice.

**FIGURE 2:**
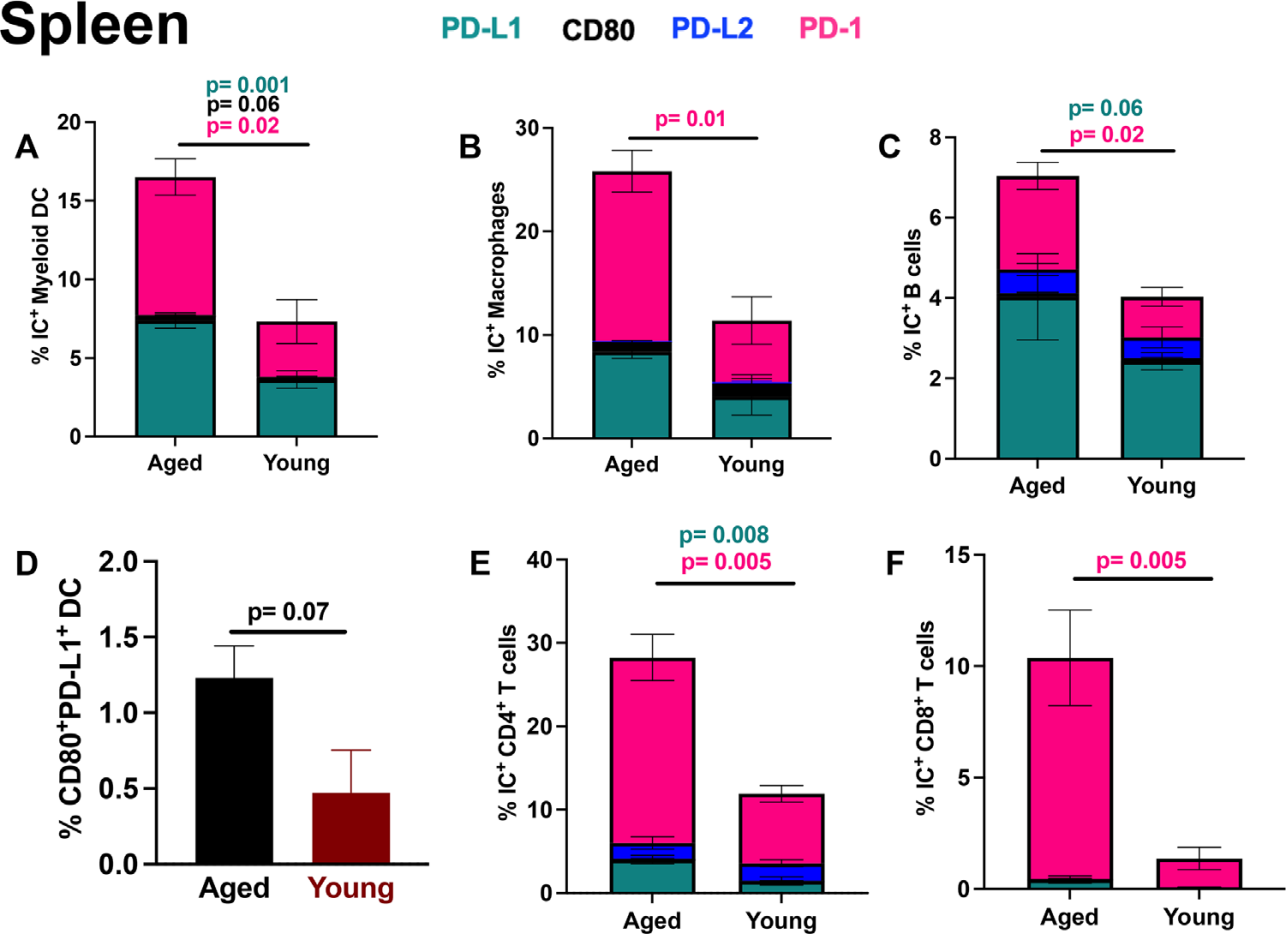
Tumor naïve aged mice have increased splenic immune cell PD-1 and PD-L1 expression versus young. Flow cytometry data on IC expression in aged versus young mice. (A–E) wild-type aged (23–26 months) and wild-type young mice (2–4 months) IC expression prevalence in spleen. Gating scheme is (A–E) Live CD45^+^ for all cells, then (A) CD3^−^B220^−^CD11c^+^ for myeloid dendritic cells (DC), (B) CD3^−^B220^−^CD11b^+^F4/80^+^ for macrophages, (C) CD3^−^B220^+^ for B cells, (D) CD80/PD-L1 co-expression on myeloid DC (CD3^−^B220^−^CD11c^+^), (E) CD3^+^B220^−^CD8^−^ CD4^+^, and (F) CD3^+^B220^−^CD4^−^ CD8^+^. Standard error of mean indicated; *P*-values, Student’s *t*-test. *n* = 3–6 mice/group

Myeloid cell, notably dendritic cell (DC) PD-L1 expression improves *α*PD-L1 ICI efficacy,^31^ but aged mice had higher splenic DC PD-L1 expression (Figure 2A) versus young. Macrophage (Figure 2B) and B cell (Figure 2C) PD-L1 expression, which could be immunosuppressive, was also higher in the spleens of aged versus young mice, showing broad increases in PD-L1 in aged mice that could counteract potentially beneficial DC PD-L1 expression (Figure 2A). We assessed IC mean fluorescence intensity (MFI) an assessment of intensity of IC expression on a per-cell basis, as opposed to percentage (prevalence) of IC-expressing cells. In the spleen, there was increased DC PD-L1 MFI in aged versus young, but PD-L1 was also increased in total CD11b^+^ myeloid cells and total CD3^+^ T cells, further corroborating broad immune cell increase in PD-L1 in spleens of naïve aged versus young mice (Figure S1A).

PD-L1 can bind either PD-1 or CD80. CD80 binding to PD-L1 can suppress T cell functions.^32–36^ In contrast, recent studies have shown that *cis* interaction of PD-L1 and CD80 on antigen presenting cells can improve T cell responses.^37,38^ Aged splenic DC had higher CD80 expression prevalence and MFI versus young (Figures 2A and S1B). To test potential roles in *α*PD-L1 failure in aged, we assessed DC co-expression of PD-L1 and CD80. Aged splenic DCs also had higher co-expression of PD-L1 and CD80 versus young (Figure 2D). Higher DC PD-L1/CD80 co-expression on aged DC could correlate with more *cis* interaction between PD-L1 and CD80 and improve T cell responses therefore PD-L1 blockade could be detrimental and help explain lack of *α*PD-L1 efficacy in aged.

PD-1 expression on tumor-associated macrophages inhibits their phagocytosis, including of tumor cells and can dampen antitumor immunity by engaging PD-L1^+^ T cells.^39^ Additionally, myeloid cell-specific PD-1 deletion can improve antitumor immunity by preventing generation of deleterious and immunosuppressive myeloid derived suppressive cells (MDSC).^40^ Splenic DC (Figure 2A) and macrophages (Figure 2B) in aged mice showed significantly higher PD-1 expression prevalence and MFI (Figure S1C) versus young, which could indicate higher immunosuppressive potential of myeloid cells in tumor naïve aged mice versus young and help explain *α*PD-1 over *α*PD-L1 efficacy in aged.

B cells can act as antigen presenting cells and have low basal IC expression that can be upregulated upon activation.^41^ While tumor-infiltrating B cells have been studied, their function within the tumor microenvironment (TME) is unclear with some studies suggesting tumor-promoting effects, while others show that they can improve cancer outcomes.^42–45^ Splenic B cells had higher PD-L1 and PD-1 expression prevalence in aged versus young (Figure 2B). Splenic B cell CD80 prevalence trended higher in young versus aged mice (Figure 2B). In contrast, CD80 MFI was significantly higher in aged versus young B cells (Figure S1B). More work is required to understand any functional consequences of these B cell IC expression differences.

PD-L2 is also a PD-1 ligand^22^ and has only recently been widely appreciated as potentially consequential to cancer ICI outcomes. A recent study showed that in young hosts, macrophage PD-L2 expression was upregulated following *α*PD-L1 ICI and combining *α*PD-L1 with *α*PD-L2 improved anti-tumor immunity.^46^ To investigate PD-L2, we found that splenic DC (Figure 2A), macrophage (Figure 2B) PD-L2 expression prevalence, and MFI (Figure S1D) were similar in young and aged tumor-naïve mice.

T cell PD-1 can mark exhausted cells with decreased functionality versus PD-1^−^ (generally non-exhausted) counterparts.^47^ In cancer and chronic infection models, PD-1 expressing T cells are most responsive to *α*PD-L1 and *α*PD-1 ICI.^48,49^ In contrast, high levels of T cell PD-1 expression could generate *α*PD-1 resistance.^50^ We saw significantly increased PD-1 expression prevalence on splenic CD4^+^ (Figure 2E) and CD8^+^ T cell (Figure 2F) subsets and higher splenic PD-1 MFI on aged versus young T cells (Figure S1C), consistent with reports from us and others.^51,52^

T cell CD80 can interact with PD-L1 to inhibit T cell functions in tolerance models,^35^ but such effects are not reported in ICI to our knowledge. Aged CD3^+^ total T cells had significantly higher CD80 expression versus young (not shown) with a similar trend in CD4^+^ (Figure 2E) but not CD8^+^ T cell subsets (Figure 2F), suggesting that T cell CD80 expression prevalence could be a factor in reduced T cell responses in the aged.

PD-L1 and PD-L2 expression are regulated distinctly^53^ and while PD-L1 is expressed in a variety of cell types, PD-L2 expression is more restricted.^54^ T cell PD-L1 and PD-L2 function has also been little reported. Tumor-infiltrating PD-L1^+^ T cells have diverse immunosuppressive effects within the TME through both inhibitory PD-L1 signaling into CD4^+^ and CD8^+^ T cells and PD-1 engagement on other tumor-infiltrating immune cells.^55^ We found that splenic CD4^+^ (Figure 2E) but not CD8^+^ T cell (Figure 2F) subsets in aged versus young had higher PD-L1 expression prevalence, whereas PD-L2 expression prevalence and MFI (Figure S1D) were similar.

To understand if IC expression changes were also present in primary lymphoid organs of aged versus young mice, such as BM and thymus where immune cells initially develop^56^ versus the secondary lymphoid organ, spleen, we measured immune cell IC expression in them. BM DC from young mice trend toward higher PD-L1 expression (Figure S2A), while total myeloid cells and macrophages had similar PD-L1 expression (Figure S2A). Total myeloid cell and macrophage CD80 expression was similar in BM of aged versus young (Figure S2B). In contrast, CD80 expression on BM DC trended higher in aged versus young (Figure S2B). BM myeloid cells had similar PD-1 expression prevalence in aged versus young (Figure S2C). PD-L2, the second PD-1 ligand, had low expression on BM myeloid cells and was similar in aged versus young mice (Figure S2D). Generally, there were only minor changes in IC expression in BM myeloid cells in young versus aged.

**FIGURE 3:**
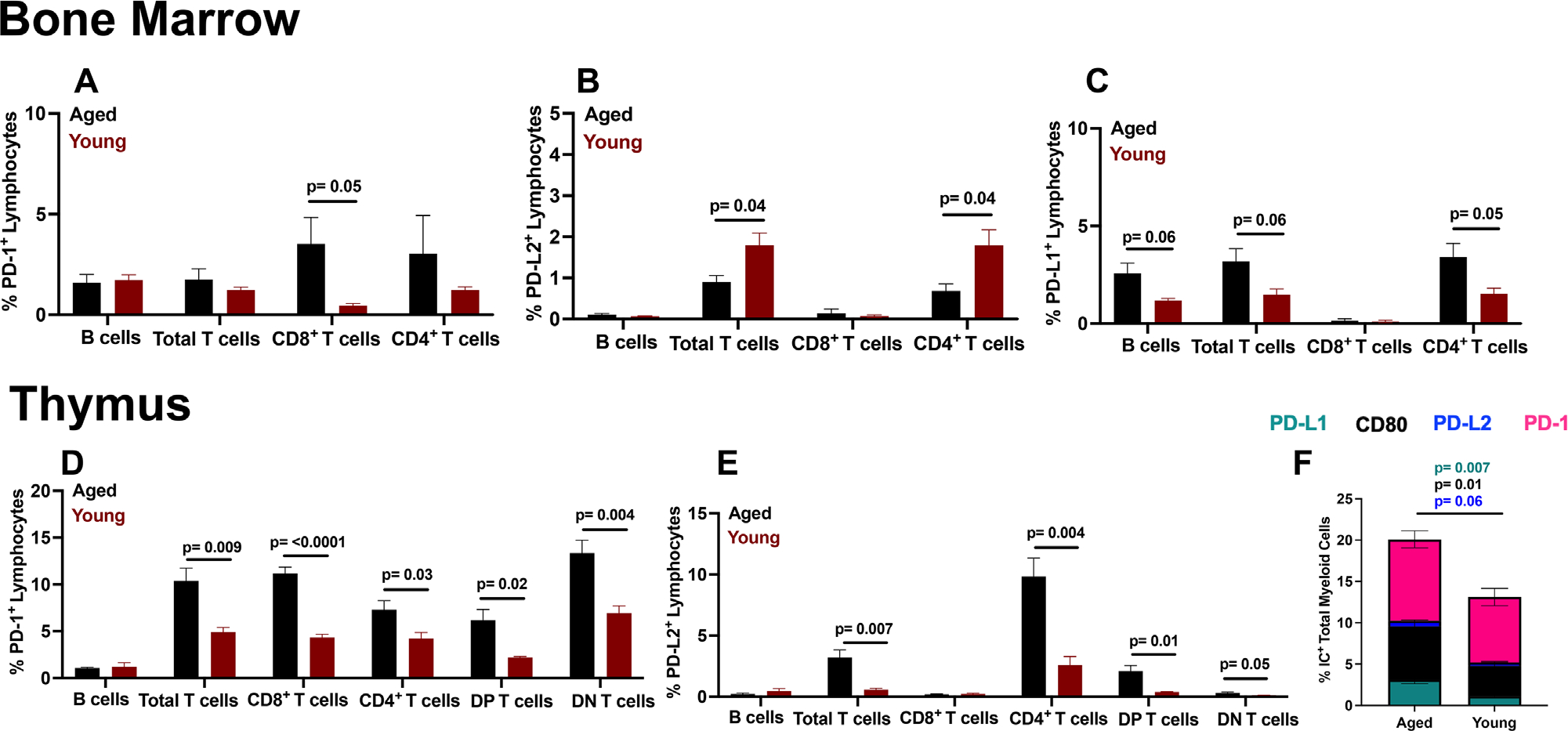
Differential immune checkpoint (IC) expression in primary lymphoid organs of aged versus young tumor naïve mice. Flow cytometry data on immune checkpoint expression in aged versus young mice. (A–F) wild-type aged (23–26 months) and wild-type young mice (2–4 months) percent IC expression data from (A–C) bone marrow, (D–F) thymus Gating scheme for A–F is as follows: live CD45^+^ for all cells, then CD3^−^B220^+^ for B cells, CD3^+^B220^−^ and either CD8^−^CD4^+^, CD4^−^CD8^+^, CD4^+^CD8^+^ (double positive, DP) or CD4^−^CD8^−^ (double negative, DN) for T cells and CD3^−^B220^−^CD11b^+^ for total myeloid cells. Standard error of mean indicated; *P*-values, Student’s *t*-test. *N* = 3–6 mice/group

Blocking PD-L1 on DC or PD-1 on T cells can improve T cell priming and promote anti-tumor immunity.^57^ As priming of T cells with tumor-associated antigens could occur in BM,^58^ which is the site of B cell development,^59^ we surveyed its T and B cells to find similarities but also notable differences in young versus aged, (Figures 3A–C and S2A–H).

The thymus is the site of T cell maturation and undergoes involution with age, which reduces adaptive immunity potential.^60^ As consequences of thymic immune cell IC in cancer immunotherapy are largely undefined, we report some results here (Figures 3D–F and S3).

**FIGURE 4:**
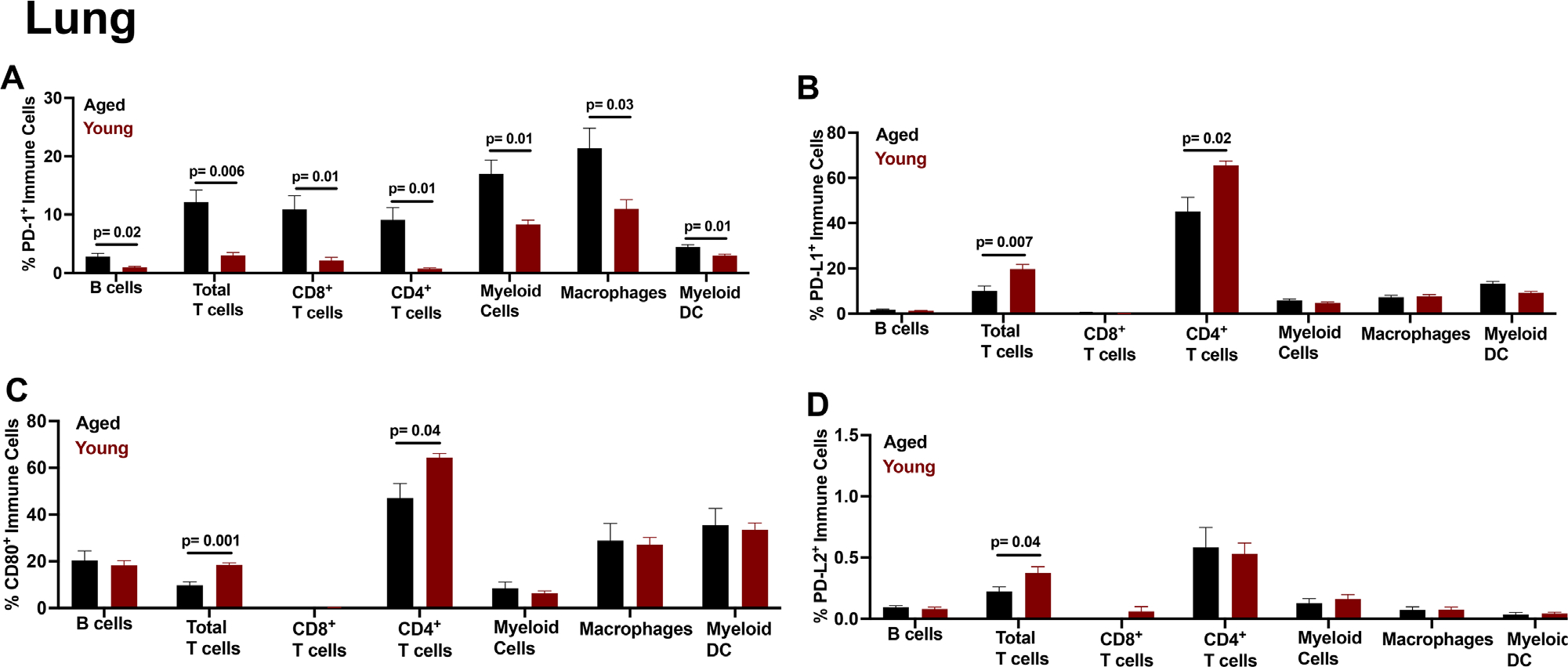
Tumor naïve aged mice exhibit increased lung immune cell PD-1 expression versus young. Flow cytometry data on immune checkpoint expression in aged versus young mice. (A–D) wild-type aged (23–26 months) and wild-type young mice (2–4 months) IC expression prevalence in lung. (A–D) Gating scheme is live CD45^+^ for all cells, then CD3^−^B220^+^ for B cells, CD3^+^B220^−^ for total T cells, CD3^+^B220^−^CD8^−^ CD4^+^ or CD3^+^B220^−^CD4^−^ CD8^+^ for T cells, CD3^−^B220^−^CD11b^+^ for total myeloid cells, CD3^−^B220^−^CD11b^+^F4/80^+^ for macrophages, and CD3^−^B220^−^CD11c^+^ for myeloid dendritic cells (DC). Standard error of mean indicated; *P*-values, Student’s *t*-test. *n* = 3–6 mice/group

Lung tissue harbors many immune cells, and is a site of metastasis for B16F10 murine^61^ and human melanoma.^62,63^ IC expression changes with age could influence metastatic tumor spread or ICI response there. Lung myeloid, including macrophages and DC had higher PD-1 expression prevalence (Figure 4A) and MFI (Figure S4A) in aged versus young. PD-L1 expression prevalence (Figure 4B) and MFI (Figure S4B) was also higher in aged DC versus young. Total lung CD3^+^ T cells, and CD8^+^ and CD4^+^ T cell subsets had higher PD-1 expression prevalence (Figure 4A) and MFI (Figure S4A) in aged versus young. In contrast, total lung CD3^+^ T cells and CD4^+^ T cells had lower PD-L1 (Figure 4B) and CD80 expression prevalence (Figure 4C) in aged versus young. Lung B cells had higher PD-1 expression prevalence (Figure 4A) and MFI (Figure S4A), but similar PD-L1, CD80, and PD-L2 expression prevalence (Figure 4B–D) and MFI (Figure S4B–D) in aged versus young. Gating schemes for immune cell populations from naïve mouse spleen (Figure S5), BM (Figure S6), thymus (Figure S7), and lung (Figure S8) are shown.

### 3.3 | Host IFN-*γ* alters T cell IC expression in an age- and tissue-specific manner

**FIGURE 5:**
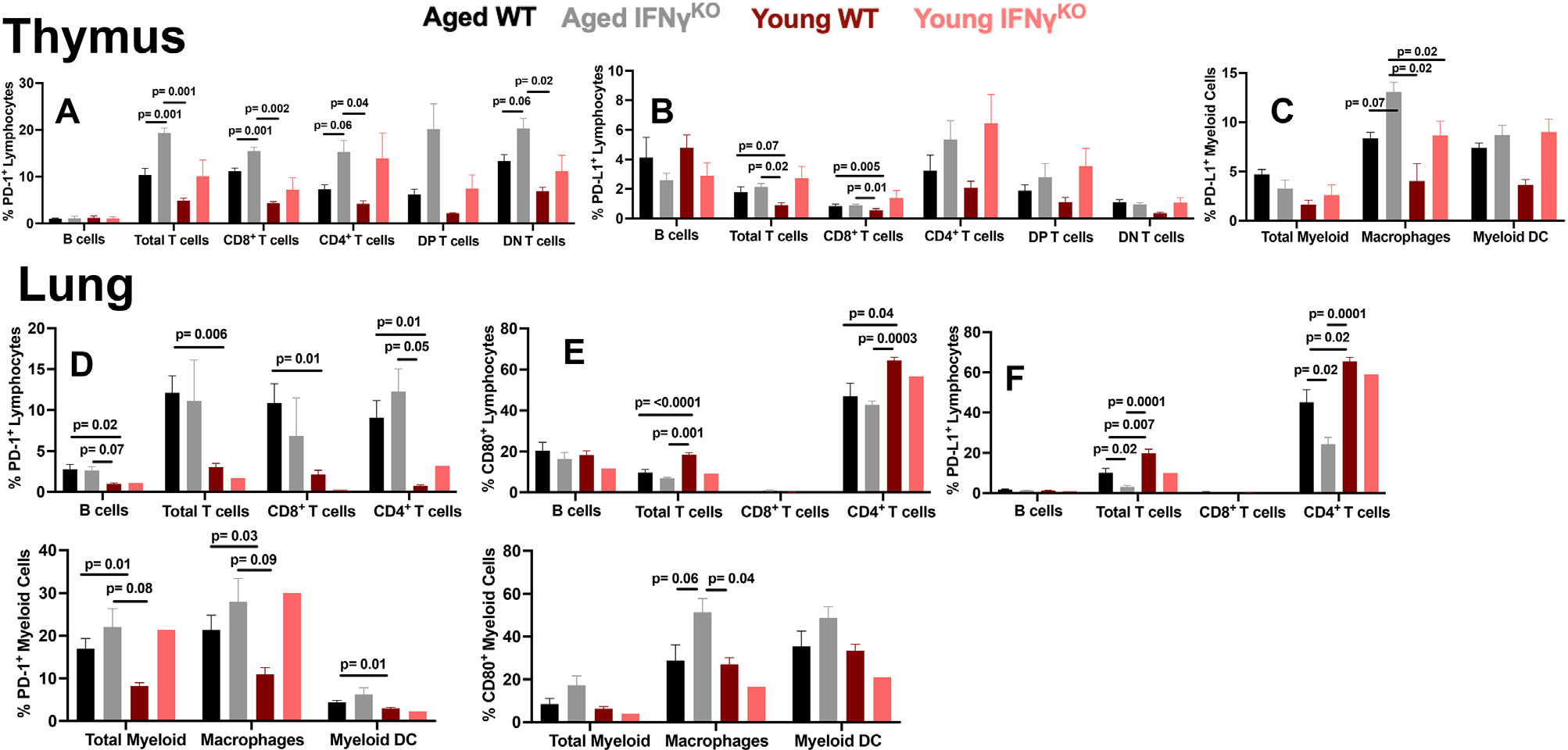
Host IFN*γ* dampens age-related increase in T cell PD-1 and PD-L1 in thymus but not lung and increases T cell PD-L2 expression in lung. Flow cytometry data on IC expression prevalence in aged wild type versus IFN*γ*^KO^ mice. (A–F) wild-type aged (23–26 months) and IFN*γ*^KO^ aged (24–28 months) IC expression prevalence (A–C) thymus and (D–F) lung (A–F) Gating scheme is live CD45^+^ for all cells CD3^−^B220^+^ for B cells, CD3^+^B220^−^ and either CD4^+^CD8^−^, CD8^+^CD4^−^, CD4^+^CD8^+^ (DP) or CD4^−^CD8^−^ for T cells, CD3^−^B220^−^CD11b^+^ for myeloid cells and CD3^−^B220^−^CD11b^+^F4/80^+^ for macrophages, CD3^−^B220^−^CD11c^+^ for myeloid dendritic cells (DC). Standard error of mean indicated; *P*-values, Student’s *t*-test. *n* = 3–6 mice/group except for young IFN*γ*^KO^ in the lung where *n* = 1

As PD-L1, PD-L2, and CD80 expression can be upregulated by IFN-*γ*, among other cytokines,^41^ and we reported that serum IFN-*γ* increases with age,^51^ we assessed host IFN-*γ* contributions to age-related IC changes in WT versus syngeneic IFN-*γ*^KO^ mice (Figure 5). As IFN-*γ*^KO^ mice are prone to cancers and infections with age, we inspected them carefully for signs of infections or obvious tumors at necropsy, and only reported evidently healthy mice for these studies. Surprisingly, we did not observe major IC expression differences between WT and IFN-*γ*^KO^ mice in spleen or BM, or significant differences in MFI of these IC (not shown), but found differences in the thymus (Figure 5A–C) and lung (Figure 5D–F).

T cell PD-1 expression prevalence in lung did not exhibit an age effect between IFN-*γ*^KO^ and WT (Figure 5D). T cell CD80 was not appreciably expressed on CD8^+^ T cells irrespective of age or IFN-*γ* competence. CD4^+^ T cell CD80 expression prevalence was lower in age, but unaffected by IFN-*γ* competence (Figure 5E).

Host IFN-*γ* did not alter PD-1 (Figure 5D, bottom), PD-L1, or PD-L2 (not shown) expression prevalence on lung myeloid cells including macrophages and DC versus aged WT. In contrast, lack of host IFN-*γ* unexpectedly increased CD80 expression prevalence in aged lung macrophages versus aged and young WT mice (Figure 5E, bottom). Gating schemes for immune cell populations of naïve mouse data are in Figure S5–8.

### 3.4 | Immune cell tumor infiltration and intra-tumoral IC expression differs in aged versus young mice

**FIGURE 6:**
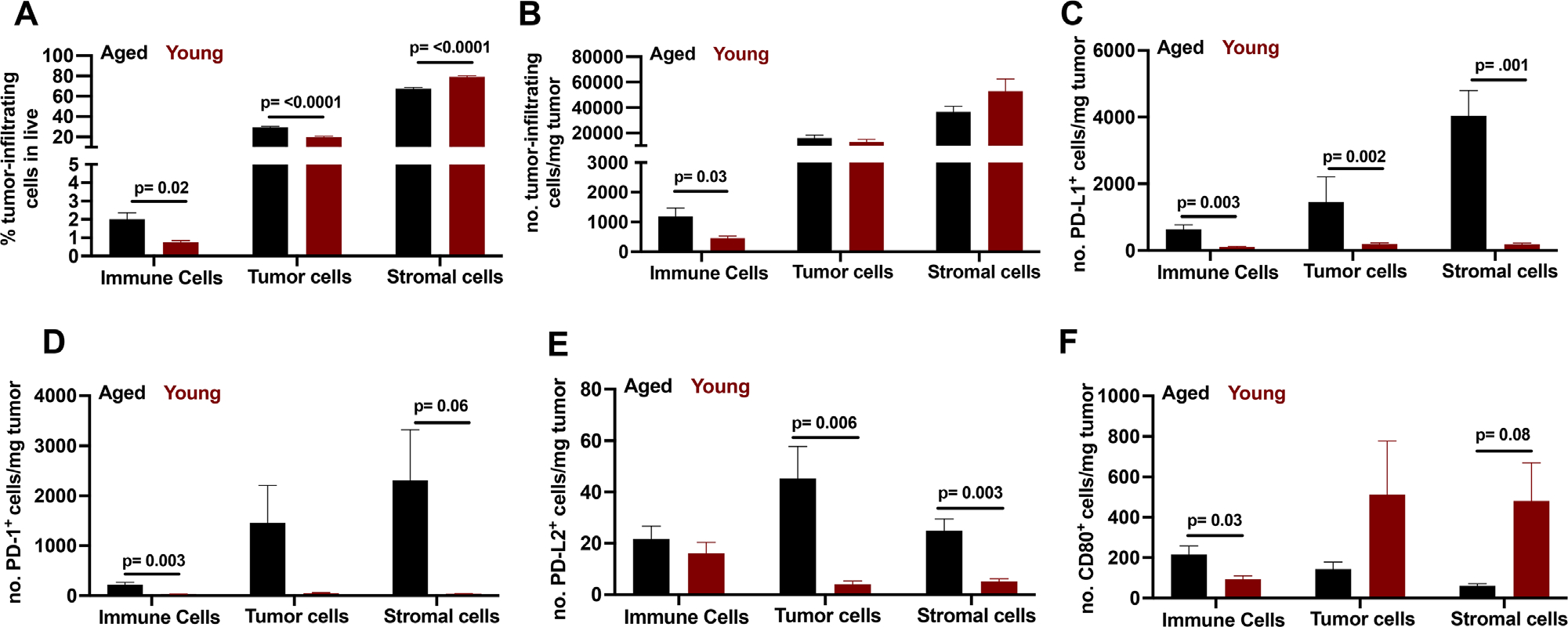
Immune cell infiltration and immune cell PD-1 and PD-L1/L2 ligand expression differs by age. Flow cytometry data on tumor-infiltrating immune cell content and IC expression prevalence in aged versus young mice. (A–F) Wild-type aged (23–26 months) and wild-type young mice (2–4 months). (A) Tumor infiltrating immune cells prevalence, (B) number tumor infiltrating immune cells, (C) number PD-L1^+^ tumor-infiltrating cells, (D) number PD-1^+^ tumor-infiltrating cells, (E) number PD-L2^+^ tumor-infiltrating cells, and (F) number CD80^+^ tumor-infiltrating cells. (A–F) Cell population gating is live CD45^+^ for immune cells, live CD45^−^SSC^hi^ for tumor cells and live CD45^−^SSC^lo^ for stromal cells. (A–F) Number (no.) cells normalized per mg of tumor. Standard error of mean indicated; *p*-values, Student’s *t*-test. *n* = 6–9 tumors/group

Tumors are controlled immunologically by infiltrating anti-tumor CD45^+^ immune cells, notably CD8^+^ T cells and their IC expression, among other factors.^64^ To understand tumor microenvironmental effects on IC expression with age, we challenged young and aged mice with B16F10 melanoma cells subcutaneously. The tumor mass contains tumor, stromal, and immune cells. We identified immune cells as CD45^+^ cells, B16F10 cancer cells (herein “B16”) as CD45^−^ side scatter (SSC)^hi^ cells, and other non-immune tumor infiltrating cells (here defined as stroma) as CD45^−^SSC^lo^ cells by flow cytometry. Aged mice had higher prevalence (Figure 6A) and concentration (cells per milligram of tumor; Figure 6B) of tumor infiltrating CD45^+^ total immune cells despite similar tumor growth velocity versus young mice (Figure 1E). Tumor masses in aged mice had a higher prevalence of B16 tumor cells and lower prevalence of stromal cells versus young mice (Figure 6A). Changes in B16 tumor cell prevalence versus stromal cells could affect tumor growth, metastatic potential,^65^ and ICI efficacy.^66^

As *α*PD-L1 ICI therapy can be influenced by tumor, stromal cell, and/or immune cell PD-L1 expression,^66^ we compared PD-L1 expression in tumors in young versus aged. Tumor masses in aged mice had higher PD-L1 expression prevalence on total immune, B16 tumor and stromal cells versus young (Figure S9A). Although PD-L1 expression can predict treatment response to *α*PD-L1 such was not the case here as there was also higher concentration of PD-L1^+^ immune, B16 tumor, and stroma cells in tumors of aged versus young mice (Figure 6C) despite their complete lack of *α*PD-L1 response (Figure 1).

Tumor masses in aged mice had higher PD-1 expression prevalence among immune, B16 and stromal cells versus tumors in young mice (Figure S9B) and higher concentration of PD-1^+^ immune and stromal cells (Figure 6D). The concentration of PD-1^+^B16 tumor cells trended higher in tumors of aged versus young mice (Figure 6D), without reaching statistical significance. B16 tumor and stromal cells but not total tumor-infiltrating immune cells exhibited higher PD-L2 expression prevalence versus young mice (Figure S9C). There was more PD-L2 positive B16 tumor and stromal cells (Figure 6E), and higher PD-L2 MFI (Figure S9E) in tumors of aged versus young mice. PD-L2 MFI (but not expression prevalence or concentration) was higher on tumor-Infiltrating immune cells of aged versus young mice (Figure S9E). Differences in tumor PD-L2 expression prevalence and higher PD-1 expression prevalence could help explain better *α*PD-1 efficacy versus *α*PD-L1 in aged mice, as *α*PD-1 potentially blocks both these inhibitory signals (from PD-L1 and PD-L2). Other differences in PD-1, PD-L1, PD-L2, and CD80 expression within tumors of young versus aged can be found in Figure 6F–H and S9A–H.

**FIGURE 7:**
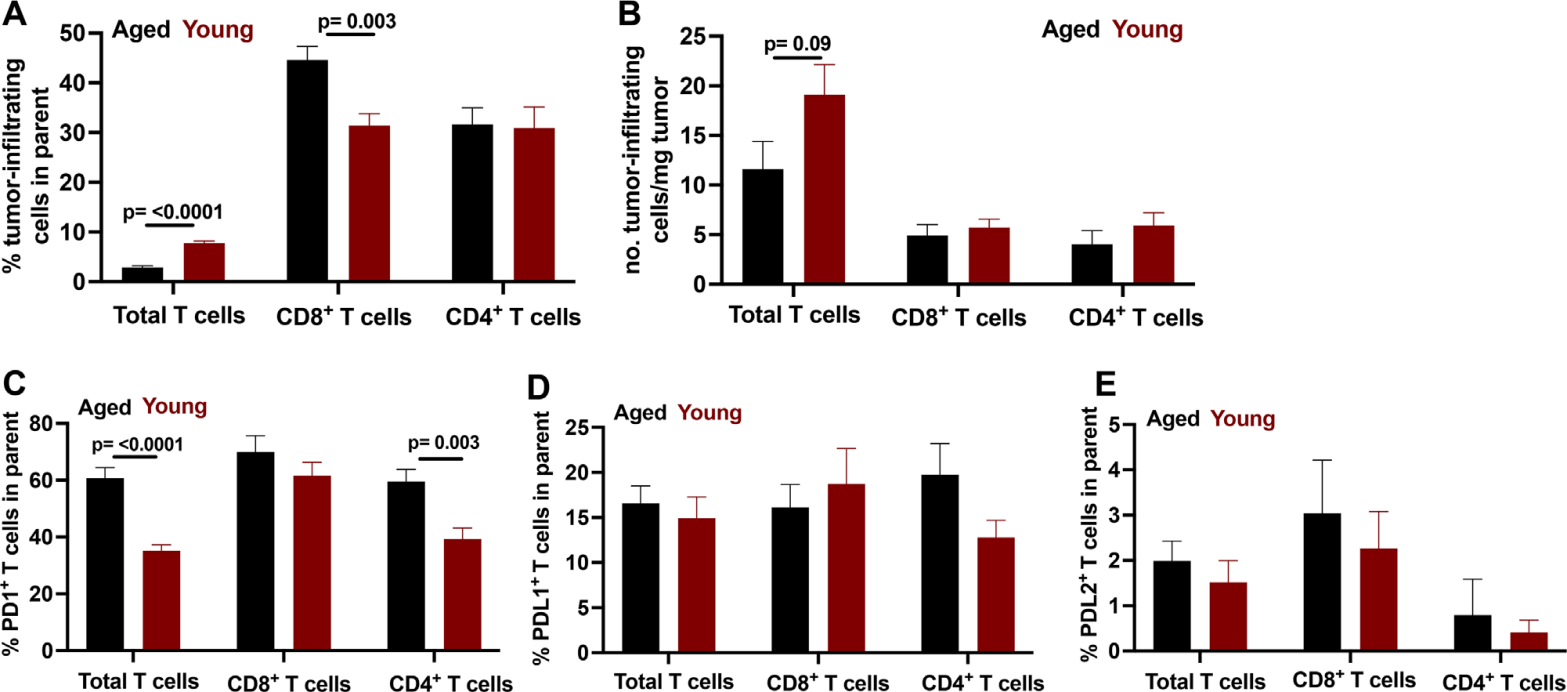
PD-1, but not PD-L1 or PD-L2 expression on tumor-infiltrating CD4^+^ T cells increases with age. Flow cytometry data on tumor-infiltrating T cells and T cell IC expression prevalence in aged versus young mice. (A–E) wild-type aged (23–26 months) and wild-type young mice (2–4 months). (A) Percent of tumor infiltrating T cells, (B) number of tumor infiltrating T cells normalized/mg of tumor, (C) PD-1 expression prevalence, (D) PD-L1 expression prevalence, and (E) PD-L2 expression prevalence. (A–E) Population gating is CD45^+^B220^−^CD3^+^ for total T cells, of CD3^+^ delineation CD4^+^CD8^−^ or CD8^+^CD4^−^. Standard error of mean indicated; *p*-values, Student’s *t*-test. *n* = 6–9 tumors/group

To assess IC expression changes in the CD45^+^ tumor-infiltrating population further, we assessed tumor-infiltrating T cell (Figure 7A–E) IC concentration and MFI on tumor-infiltrating T cells (Figure S10A–F).

**FIGURE 8:**
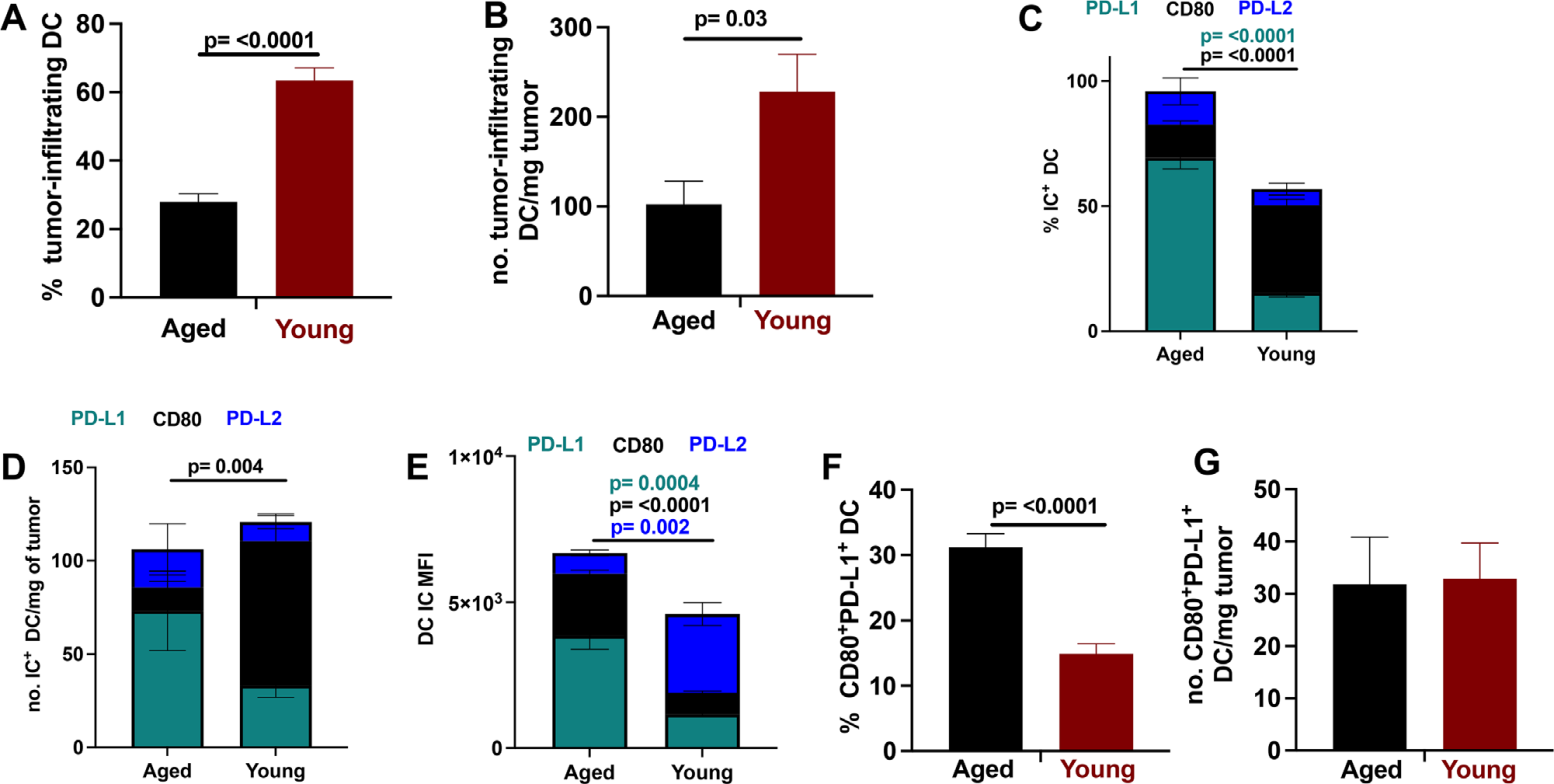
PD-L1 and PD-L2 expression on tumor-infiltrating DC increases with age. Flow cytometry data on tumor-infiltrating dendritic cells (DC), IC expression prevalence and mean fluorescence intensity (MFI) in aged versus young mice. (A–G) wild-type aged (23–26 months) and wild-type young mice (2–4 months). (A) Percent of tumor infiltrating DC, (B) number tumor infiltrating DC normalized per mg of tumor, (C) IC expression prevalence, (D) number IC^+^ tumor infiltrating DC normalized per mg of tumor, (E) DC MFI, (F) PD-L1/CD80 co-expression on DC, and (G) number CD80/PD-L1 co-expressing DC normalized per mg of tumor. (A-G) Population gating is CD45^+^B220^−^CD3^−^CD11c^+^ for DC. Standard error of mean (SEM) indicated; *p*-values, Student’s *t*-test. *n* = 6–9 tumors/group

Young mice had a higher prevalence (Figure 8A) and number (Figure 8B) of tumor-infiltrating DC versus aged, but aged tumor-infiltrating DC had significantly higher CD80 and PD-L1 expression prevalence (Figure 8C). Tumors in aged mice had significantly a higher number of CD80 expressing DC, that also trended to higher PD-L1 and PD-L2 expression prevalence versus tumors in young mice (Figure 8D). IC MFI on tumor-infiltrating DC showed similar trends except for significantly higher PD-L2 MFI on tumor-infiltrating DC in young versus aged (Figure 8E). We also assessed co-expression of PD-L1 and CD80 on DCs, as our data on naïve mice showed higher co-expression in aged that could help explain lack of *α*PD-L1 efficacy. DCs from tumors in aged mice had higher PD-L1 and CD80 co-expression prevalence versus young (Figure 8F), similar to aged mouse spleen. Total number of PD-L1/CD80 co-expressing DC was similar in tumors of aged versus young (Figure 8G) due to the higher number of tumor-infiltrating DC in tumors from young mice versus aged. Gating schemes for tumor-infiltrating cell populations are in Figure S11.

### 3.5 | *α*PD-1, *α*PD-L1, and *α*CD80 alter cytokine production distinctly in aged versus young T cells

We developed a co-culture system to interrogate age effects on myeloid cell/T cell interactions based on known importance of the former on the latter in anti-cancer immunity and immunotherapies.^67^

**FIGURE 9:**
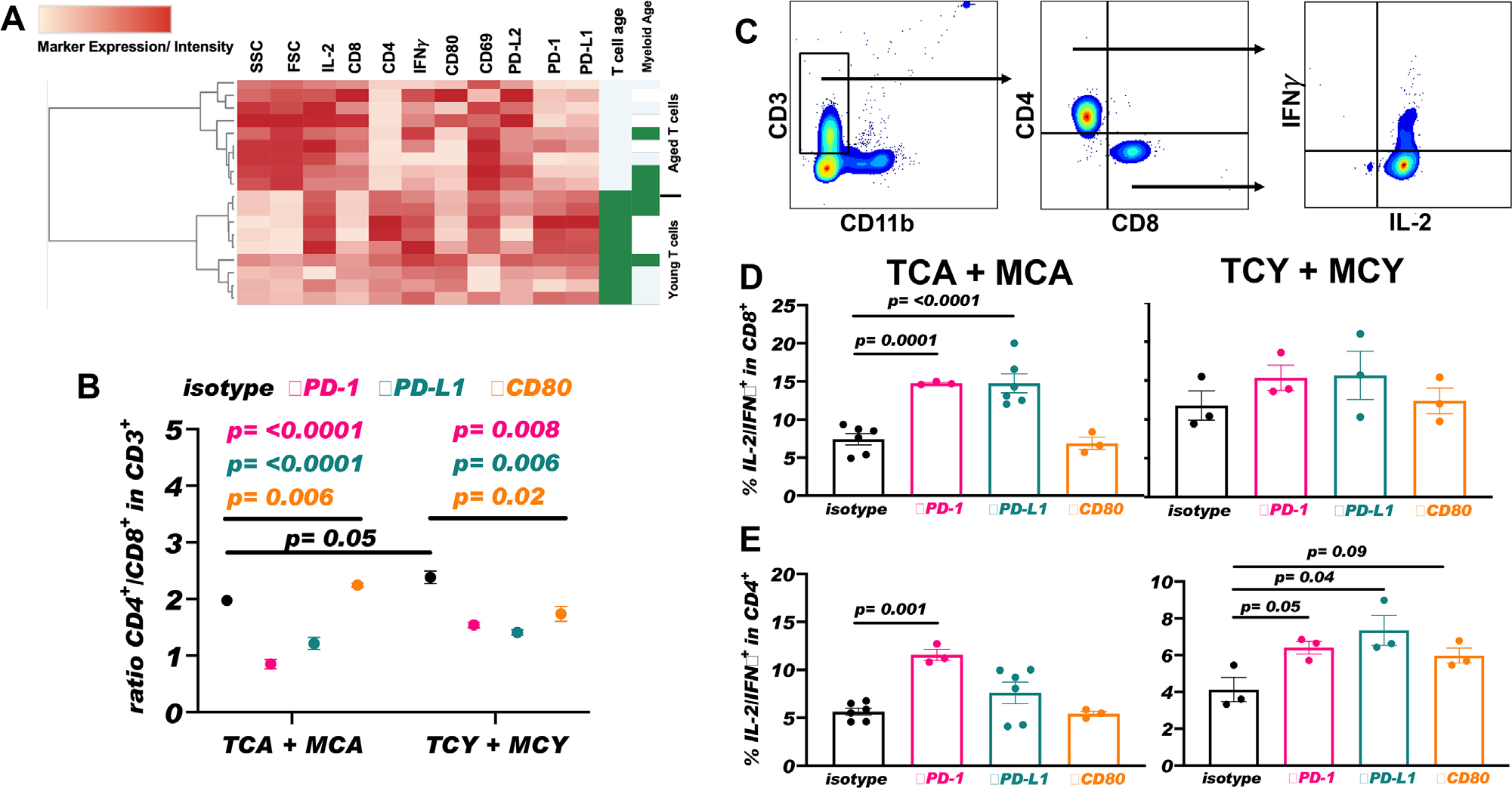
*α*PD-1, *α*PD-L1, and *α*CD80 alter cytokine production distinctly in aged versus young T cells. Analysis of co-culture flow cytometry data using OMIQ software. (A) Heatmap of flow cytometry marker expression and intensity of pre-gated co-culture T cells (gated as B220^−^CD11b^−^CD3^+^ singlets) within all co-culture conditions. (B) CD4^+^/CD8^+^ T cell ratio in each *α* treatment group (isotype control, *α*PD-1, *α*PD-L1, or *α*CD80) in aged T cells co-cultured with aged myeloid cells (TCA + MCA) or young T cells co-culture with young myeloid cells (TCY + MCY). (C) Gating scheme for cytokine (IFN*γ* and IL-2) expression prevalence in CD3^+^CD4^−^CD8^+^ or CD3^+^CD4^+^CD8^−^. (D) Prevalence of IL-2^+^IFN*γ*^+^ CD8^+^ T cells in TCA + MCA or TCY + MCY. (E) Prevalence of IL-2^+^IFN*γ*^+^ CD4^+^ T cells in TCA + MCA or TCY + MCY. Pre- and post-sort populations and gating can be found in Figure S12. Standard error of mean indicated; *p*-values, Student’s *t*-test. *n* = 1–2 biological replicates and experimental triplicates for each biological replicate

We used heatmap analysis to interrogate differences in T cell activation markers, cytokine production, and IC expression in co-cultured myeloid and T cells (Figure 9A). Co-culture of activated T cells showed that aged T cell side and forward scatter was increased, consistent with activation (Figure 9A). There were higher CD8 versus CD4 content in aged versus young T cells (Figure 9A) indicating either (1) differences in CD8^+^ versus CD4^+^ T cell prevalence within spleens of aged versus young and/or (2) higher proliferative potential of CD8^+^ versus CD4^+^ T cells in co-culture conditions with aged versus young T cells. Our post-sort data indicated that young spleens had about 20% more CD4^+^ T cells and about 30% fewer CD8^+^ T cells versus aged spleens (Figure S12A–F), indicating that there was a skewed CD4/CD8 ratio even before co-culture. Furthermore, we assessed CD4/CD8 ratio in isotype treated co-culture conditions and confirmed higher CD4/CD8 ratio in conditions with young versus aged T cells, indicating a treatment-independent skewing (Figure 9B). Heatmap co-culture results suggested that aged T cells exhibit signs of increased activation versus young independent of myeloid cell age in co-culture.

To gain insights into how ICI agents could affect aged versus young T cells differently, we treated aged T cells co-cultured with aged myeloid cells or young T cells co-cultured with young myeloid cells with *α*PD-1, *α*PD-L1, *α*CD80 antibodies, or respective isotype controls. *α*PD-1 and *α*PD-L1 decreased CD4/CD8 T cell ratio in young and aged co-culture conditions versus isotype controls (Figure 9B), consistent with supporting CD8^+^ T cell expansion, which is favorable to anti-tumor immunity. In contrast, *α*CD80 increased the CD8/CD4 T cell ratio only in young cells, of unclear significance. (Figure 9B).

We also assessed polyclonal IL-2/IFN-*γ*-producing CD4^+^ and CD8^+^ T cells (Figure 9C–D) that support anti-tumor immunity. *α*PD-1 and *α*PD-L1 significantly increased polyfunctional IL-2/IFN-*γ*-producing CD8^+^ T cells in aged with a similar, but not significant trend in young CD8^+^ T cells (Figure 9D). *α*PD-1 significantly increased polyfunctional IL-2/IFN-*γ*-producing CD4^+^ T cells in both young and aged, as did *α*PD-L1 only in young. *α*PD-1 was significantly more able to augment aged versus young polyfunctional generation. *α*CD80 had no significant effect on any T cell in young or aged. *α*PD-1 significantly improved polyfunctional T cells in both CD4^+^ and CD8^+^ subsets in aged mice, which could help explain its efficacy in them against B16F10 tumor growth, although many other factors are likely also involved.

## 4 | DISCUSSION

Many factors influence ICI efficacy, including IC expression on tumor and immune cells, tumor mutations, local immunosuppressive factors, and tumor-infiltrating immune cell content and function.^66^ Despite the impressive efficacy of ICI in some cancers and many FDA approvals including eight approved ICI antibodies, relatively little regarding age effects on ICI have been reported.

Clinical data on ICI efficacy in elderly individuals with cancer are encouraging with retrospective meta-analyses showing similar efficacy in aged (> 60 years) versus young (< 60 years) melanoma patients treated with *α*PD-1 and other ICI agents.^14–17^ Additional retrospective meta-analyses data show that elderly cancer patients (> 50 years) have a better response to ICI than younger patients (< 50 years) and patient age may be a useful biomarker in determining response to ICI.^18^ In contrast, a recent retrospective meta-analysis showed no significant PFS improvement in cancer patients ≥ 75 years old treated with *α*PD-1 or *α*PD-L1 monotherapy or *α*PD-1 + *α*CTLA-4 combination therapy versus control groups. By contrast younger patients (< 75 years) had significant PFS improvement.^19^ Despite contradictory data, age-related differences surely occur based on a large body of data regarding age-related immune effects generally. Reasons for lack of finding a significant age effect on cancer ICI include underpowered clinical studies due to low elderly patient enrollment,^20^ combining various ICI agents rather than comprehensive analyses of individual agents, variable definitions of “aged” and the possibility that in regards to ICI, an age effect does not appear until very advanced age.

To further explore ICI efficacy in young versus aged, we studied effects in pre-clinical models. Although *α*PD-1 and *α*PD-L1 block a receptor (PD-1)/ligand (PD-L1) signal axis, we previously showed that in B16F10 melanoma, young mice responded to *α*PD-1 and *α*PD-L1 ICI, whereas *α*PD-L1 was ineffective in aged mice despite *α*PD-1 efficacy.^10^ We hypothesized that this unexpected, dichotomous outcome reflected differential, age-related IC (PD-1, PD-L1, PD-L2, CD80) expression, among other considerations as IC other than PD-1 and PD-L1 are largely not considered in IC efficacy (or age) studies.

Little is known about how IC expression changes in humans as they age. In non-elderly adult humans, PD-1 is not expressed on naïve T cells and has low expression on naïve and activated B cells and myeloid cells.^41^ Additionally, PD-L2 and PD-L1 are not expressed in naïve T cells and have low expression on myeloid cells, while no such data are reported on naïve or activated B cells.^41^ A recent report on IC expression differences between young and aged adults showed that naïve CD4^+^ T cells in aged adults have higher expression of PD-1 versus young adults.^68^ There is no data on PD-L1, PD-L2, or CD80 expression in elderly humans, to our knowledge.

We assessed age effects on PD-1, PD-L1, PD-L2, and CD80 to help support additional studies of age effects on IC efficacy, and to support age effects on other immune outcomes related to these IC pathways. We found higher PD-1 expression on many immune cells versus in young mice, which could support *α*PD-1 efficacy, but could also support *α*PD-L1 efficacy as PD-1 delivers immune inhibitory signals through ligation by PD-L1. Known age-associated PD-1 upregulation on CD8^+^ and CD4^+^ T cells, associated with increased CD8^+^ T cell exhaustion, and a decline in CD4^+^ effector memory T cell function^69,70^ could also be differentially affected by *α*PD-1 versus *α*PD-L1. For example, upregulation of PD-1 on aged T cells could influence *α*PD-1 differentially versus *α*PD-L1 ICI efficacy as PD-1 is ligated by both PD-L1 and PD-L2,^24^ while *α*PD-L1 blocks PD-L1 but not PD-L2. In this regard, we found differential PD-L2 expression in aged versus young on distinct immune cells that could influence ICI outcomes, requiring additional investigations. *α*PD-L1 efficacy also depends on tumor environment PD-L1 content and specifically DC PD-L1.^31^ Tumors can also express PD-1,^71,72^ but little is reported about consequences.

We noted increased PD-L1 on many aged versus young immune cells, including on splenic DC, and on many tumor-infiltrating cells. Thus, lack of *α*PD-L1 efficacy in aged mice could owe to the fact that DC PD-L1 or the amount specifically expressed does not support *α*PD-L1 efficacy in aged, that PD-L1 expression is required on a specific DC subset that we did not study, or some immunosuppressive factor prevents *α*PD-L1 efficacy but not *α*PD-1 efficacy. That latter scenario is plausible as PD-1 expression was high on potentially immunosuppressive myeloid cells in aged and could be blocked by *α*PD-1 but not *α*PD-L1.

CD80 is the other PD-L1 receptor whose expression in distinct anatomic compartments, especially in age, is little reported. In one report, gut mucosal DC CD80 expression was similar in young versus aged mice, but IL-15 upregulated mucosal DC CD80 expression more in aged.^73^ We noted differences in DC CD80 in various compartments studied such as increased T cell and splenic DC CD80 expression and per-cell expression (MFI). The effect was more pronounced on CD4^+^ rather than CD8^+^ T cells considered to be the major anti-tumor T cells. Nonetheless, this high CD80 prevalence and/or per-cell expression could reduce *α*PD-L1 efficacy in aged versus young through mechanisms that would not directly affect response to *α*PD-1. For example, CD80/PD-L1 binding can produce a heterodimer that prevents PD-1 ligation but maintains CD80/CD28 co-stimulation signaling. Blocking PD-L1 in this case could dampen anti-tumor immunity.^37^ In support, we found that aged splenic DCs in tumor-naïve mice have higher co-expression of PD-L1 and CD80 versus young, which suggests a general age-related effect, but we also noted higher DC PD-L1/CD80 in tumor infiltrating DC. Higher DC PD-L1/CD80 co-expression on aged DC could help explain lack of *α*PD-L1 efficacy in aged. Further work on functional outcomes of differential IC expression is ongoing in our lab to understand ICI outcome differences. Our co-culture system (discussed further below) can be used to interrogate this pathway in detail.

We previously found that B cell PD-L2 (B7-DC) increased in spleen, blood, and BM with age, that aged PD-L2^+^ B cells regulate CD4^+^ T cell functions distinctly from young in a PD-L2-dependent manner and that PD-L2^+^ B cells inhibit subcutaneous MC38 colon cancer growth.^74^ Our current data show increased PD-L1, PD-1, and CD80 expression in aged versus young B cells but similar PD-L2 expression. Both groups of aged mice in our prior and current reports are on the BL6 background but are slightly different BL6 strains, and have distinct gut microbiomes^75^ (and our unpublished data). Prior and current mice were originally purchased from different vendors and aged in distinct facilities, among other considerations to explain these discordant data. These differences highlight variations in aged populations that can influence ICI outcomes and other age-related effects that deserve much more study.

While much has been explored regarding changes in immune cell populations in naïve and tumor-bearing mice, many gaps in knowledge remain, specifically when it comes to age-related differences. For example, decreased tumor infiltrating Tregs were associated with improved *α*PD-1 response in aged versus young mice with a subcutaneous melanoma model (YUMM). In contrast, a study on triple negative breast cancer reported diminished IFN*γ* signaling by tumors in older mice and patients that correlated with diminished response to *α*PD-1 and *α*CTLA-4 in aged versus young mice.^76^ Another report on glioblastoma brain tumors found that aged mice (> 18 months old) had decreased response to anti-PD-L1 + anti-CTLA-4 combination therapy versus younger mice (2–6 months old).^13^ T cells in aged mice with oral cancer (NR-S1K oral squamous cell carcinoma) show increased PD-1 and CTLA-4 expression versus young mice, and remarkably, *α*PD-1, *α*CTLA-4, and *α*PD-L1 resulted in better tumor control in aged versus young.^77^ In this model, aged mice responded to *α*PD-L1 in contrast to lack of its efficacy in our aged mice with melanoma here, further emphasizing the role of tumor type or microenvironment on ICI efficacy. In this regard, little is reported on differential age effects of ICI in lung metastases. Our new data on lung ICI expression will help understand poorer ICI efficacy in lung metastases versus primary tumors as we recently showed in young mice with bladder cancer.^78^

We found variably differing IC expression in thymus, lung, and BM (discussed in Supporting information) immunopathologic import of which is currently largely unknown but whose understanding can be furthered using data such as we show here.

Another significant consideration is the basis for age-related changes in the IC expression. We previously reported that cell-intrinsic mTOR promoted age-related T cell PD-1 expression and function in mouse cells^75^ and mTOR control of human T cell PD-1 expression including in the aged was recently reported.^79^ As IFN-*γ* among other cytokines can upregulate PD-L1, CD80, and PD-L2 expression,^41^ we expected host IFN-*γ* would promote IC expression as we reported that IFN-*γ* increases with age.^75^ However, we unexpectedly found that host IFN-*γ* alone did not have obvious influences on most cells in compartments studied (spleen, BM, thymus, and lung) and dampened age-related increased T cell PD-1 and PD-L1 expression in the thymus. IFN-*γ*^KO^ mice can develop compensatory programs (such as increased IL-17 or IL-4) that can confound IFN-*γ*-specific analyses, and thus additional studies of cytokine contributions to age-related IC expression are warranted.

To diminish confounding factors and assess the interaction between T and myeloid cells in young versus aged in the setting of limiting availability of aged mice, we developed a co-culture system to gain mechanistic insights, initially focused on T cells functions, not IC expression mechanisms. Our co-culture data suggested that T cell age effects were either T cell-intrinsic, or not necessarily through myeloid cells as expected. We hypothesized that myeloid cell effects would be better demonstrated if we blocked PD-1, PD-L1, or CD80 signals based on IC expression data and differential response to *α*PD-1 and *α*PD-L1 we observed in vivo. Using our co-culture system, we found that *α*PD-1 increased polyfunctional T cells in aged where *α*PD-1 was effective, and *α*PD-L1 increased beneficial CD8^+^ T cell content despite lack of treatment efficacy in vivo in aged. In contrast, *α*CD80 increased the CD4/CD8 ratio in aged co-cultures, and did not increase prevalence of polyfunctional T cells, favoring our hypothesis that increased CD80 in aged could contribute to *α*PD-L1 failure. These data show that this co-culture system can generate testable hypotheses and help define mechanisms with additional studies.

Our studies here as well as other studies focusing on the aging cancer population can further knowledge on the effects of age on immune outcomes and ICI efficacy. As we gain understanding, we can improve ICI for cancers and help make other immune agents such as vaccines more efficacious in the age populations most at risk for related illnesses.

## Supplementary Material

supplementary data

## Data Availability

We will make data available to qualified investigators upon reasonable request.
