# Supplementary material for: Immune checkpoint expression and relationships to anti-PD-L1 immune checkpoint blockade cancer immunotherapy efficacy in aged versus young mice
Source: Aging Cancer. Author manuscript; Available in PMC 2023 Mar 2. (PMC9980712; doi:10.1002/aac2.12045)
Supplement: supplementary data [file NIHMS1872562-supplement-supplementary_data.pdf]

Sup. Fig. 1

Spleen

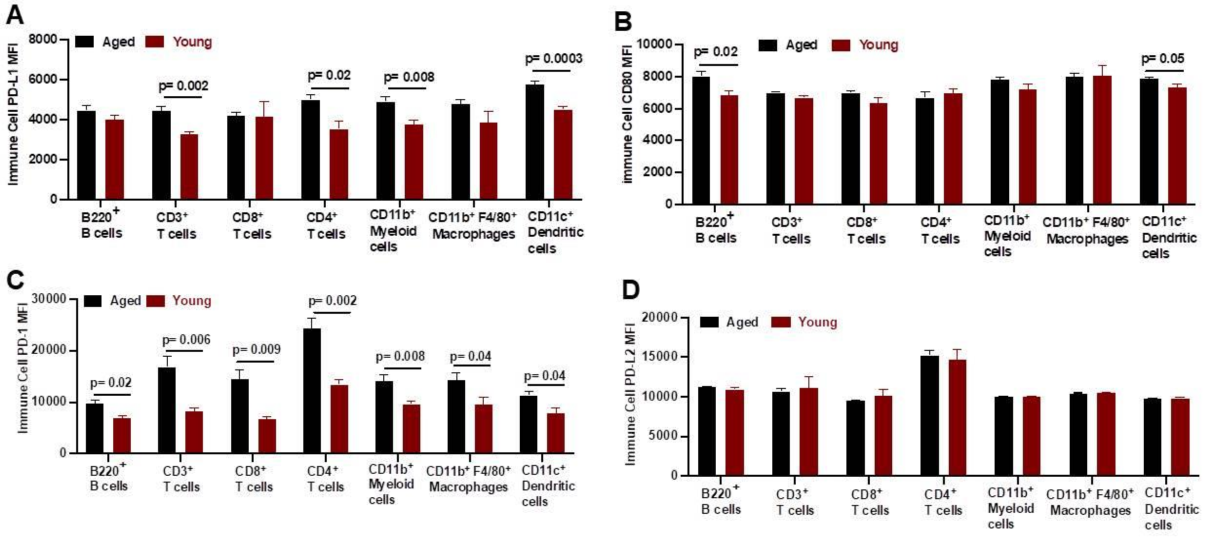

Sup. Fig. 1 Splenic IC MFI from tumor-naïve aged mice versus young mice

Flow cytometry data on splenic IC MFI (mean fluorescence intensity) in aged (23-26 months) versus young (2-4 months) mice. **(A-D)** wild-type aged and wild-type young mice **(A)** PD-L1 MFI **(B)** CD80 MFI and **(C)** PD-1 MFI and **(D)** PD-L2 MFI. **(A-D)** Gating scheme is live $CD45^+$ cells and then $CD3^-B220^+$ for B cells, $CD3^+B220^-$ and either $CD8^-CD4^+$ or $CD4^-CD8^+$ for T cells, $CD3^-B220^-CD11b^+$ for myeloid cells, $CD3^-B220^-CD11b^+F4/80^+$ for macrophages and $CD3^-B220^-CD11C^+$ for dendritic cells. Standard error of mean indicated; *p*-values, Student's *t*-test. N= 3-6 mice/group.

Sup. Fig. 2

Bone Marrow

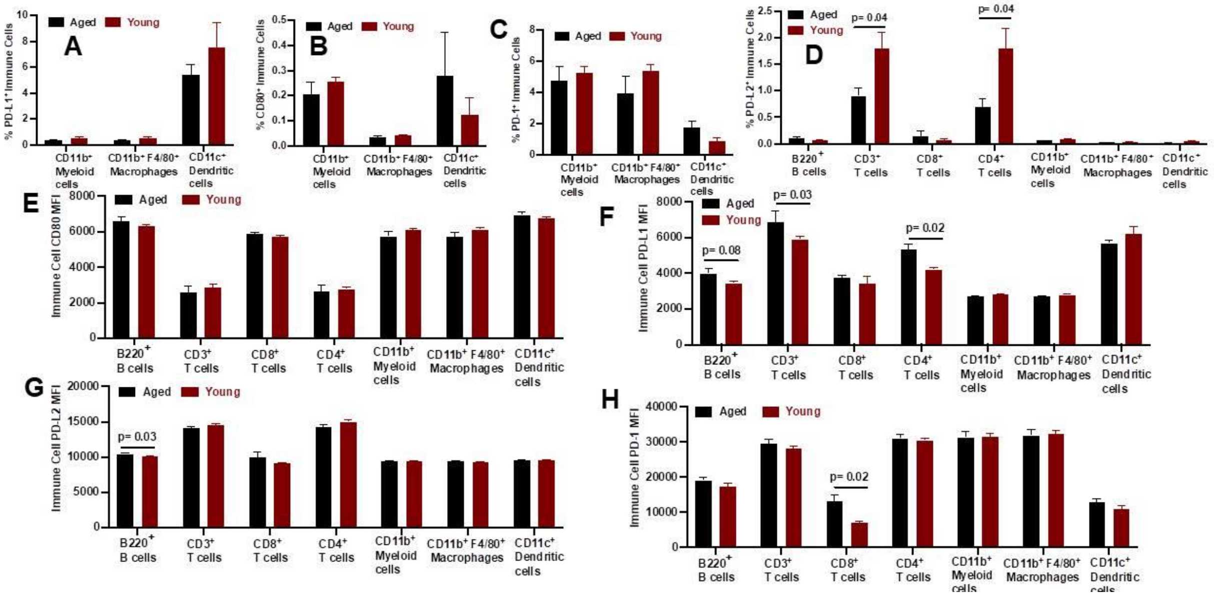

Sup. Fig. 2 Bone marrow IC expression and MFI in tumor-naïve aged mice versus young mice

Flow cytometry data on IC expression prevalence and MFI (mean fluorescence intensity) in aged (23-26 months) versus young (2-4 months) mice. (**A-H)** wild-type aged and wild-type young mice immune checkpoint expression data from **(A)** % CD80$^+$ immune Cells **(B)** % PD-L1$^+$ immune cells **(C)** % PD-L2$^+$ immune cells **(D)** % PD-1$^+$ immune cells **(E)** CD80 MFI **(F)** PD-L1 MFI **(G)** PD-L2 MFI **(H)** PD-1 MFI **(A-G)** Gating scheme is live CD45$^+$ cells and then CD3$^-$B220$^+$ for B cells, CD3$^+$B220$^-$ and either CD8$^-$CD4$^+$ or CD4$^-$CD8$^+$ T cells, CD3$^-$B220$^-$CD11b$^+$ for myeloid cells, CD3$^-$B220$^-$CD11b$^+$F4/80$^+$ for macrophages and CD3$^-$B220$^-$CD11c$^+$ for dendritic cells. Standard error of mean indicated; *p*-values, Student's *t*-test. N= 3-6 mice/group.

Sup. Fig. 3

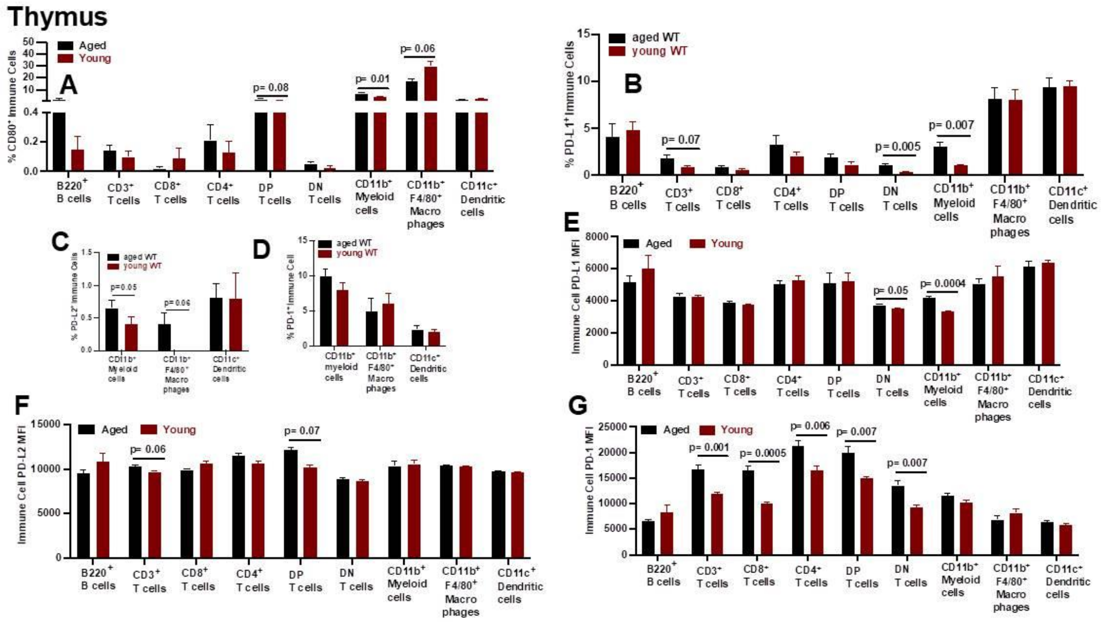

Sup. Fig. 3 Thymic IC expression and MFI in tumor-naïve aged mice versus young mice

Flow cytometry data on immune checkpoint expression prevalence and MFI (mean fluorescence intensity) in aged (23-26 months) versus young (2-4 months) mice. (**A-G)** wild-type aged and wild-type young mice immune checkpoint expression prevalence in **(A)** $CD80^+$ immune cells **(B)** PD-$L1^+$ immune cells **(C)** PD-$L2^+$ immune cells **(D)** PD-1^+ immune cells **(E)** PD-L1 MFI **(F)** PD-L2 MFI **(G)** PD-1 MFI **(A-G)** Gating scheme is live $CD45^+$ cells and then $CD3^-B220^+$ for B cells, $CD3^+B220^-$ and either $CD8^-CD4^+$, $CD4^-CD8^+$, $CD4^+CD8^+$ (DP) or $CD4^-CD8^-$ for T cells, $CD3^-B220^-CD11b^+$ for myeloid cells, $CD3^-B220^-CD11b^+F4/80^+$ for macrophages and $CD3^-B220^-CD11c^+$ for dendritic cells. Standard error of mean indicated; *p*-values, Student's *t*-test. N= 3-6 mice/group.

Sup. Fig. 4

Lung

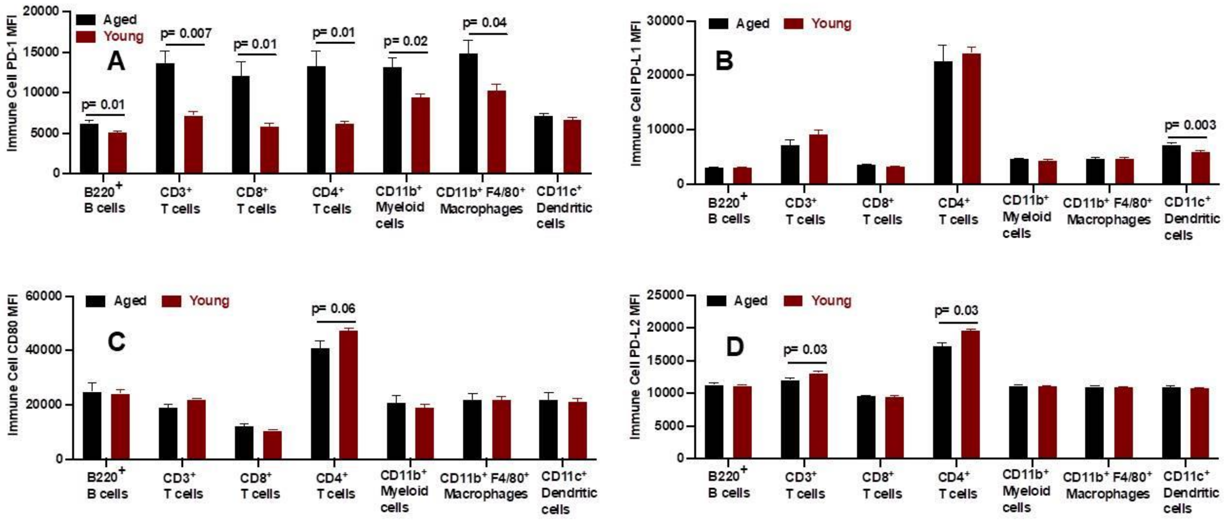

Sup. Fig. 4 Lung IC MFI from tumor-naïve aged mice versus young mice

Flow cytometry data on splenic IC MFI (mean fluorescence intensity) in aged (23-26 months) versus young (2-4 months) mice. (**A-D)** wild-type aged and wild-type young mice **(A)** PD-1 MFI **(B)** PD-L1 MFI and **(C)** CD80 MFI and **(D)** PD-L2 MFI. **(A-D)** Gating scheme is live $CD45^+$ cells and then $CD3^-B220^+$ for B cells, $CD3^+B220^-$ and either $CD8^-CD4^+$ or $CD4^-CD8^+$ for T cells, $CD3^-B220^-CD11b^+$ for myeloid cells, $CD3^-B220^-CD11b^+F4/80^+$ for macrophages and $CD3^-B220^-CD11c^+$ for dendritic cells. Standard error of mean (SEM) indicated; *p*-values, students t-test. N= 3-6 mice/group.

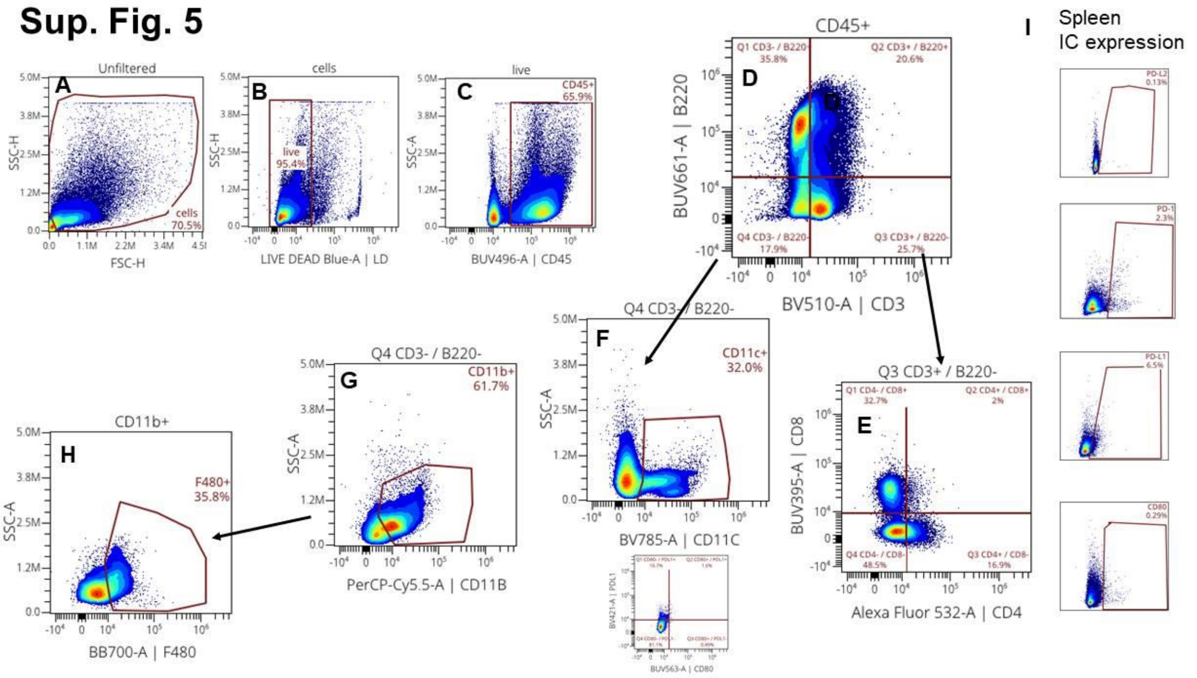

Sup. Fig. 5 Immune cell population gating scheme for spleen data from naïve mice

Gating schemes for immune cell populations depicted in Fig. 2. **(A)** Total cells **(B)** Live cells in total cell gate **(C)** CD45$^+$ cells in live cells **(D)** B220$^+$ and/or CD3$^+$ in CD45$^+$ B220$^+$CD3$^-$ were termed "B cells", B220$^-$CD3$^+$ were termed "total T cells" **(E)** CD8$^+$ and/or CD4$^+$ in B220$^-$CD3$^+$ **(F)** CD11c$^+$ in B220$^-$CD3$^-$ were termed "myeloid DC" and PD-L1/CD80 co-expression on DC **(G)** CD11b$^+$ in B220$^-$CD3$^-$ were termed "total myeloid cells" **(H)** F4/80$^+$ in CD11b$^+$ were termed "macrophages" **(I)** PD-L2, PD-1, PD-L1 and CD80 immune checkpoint expression gating **(A-I)** OMIQ software was used to analyze and gate all data. N= 3-6 mice/group.

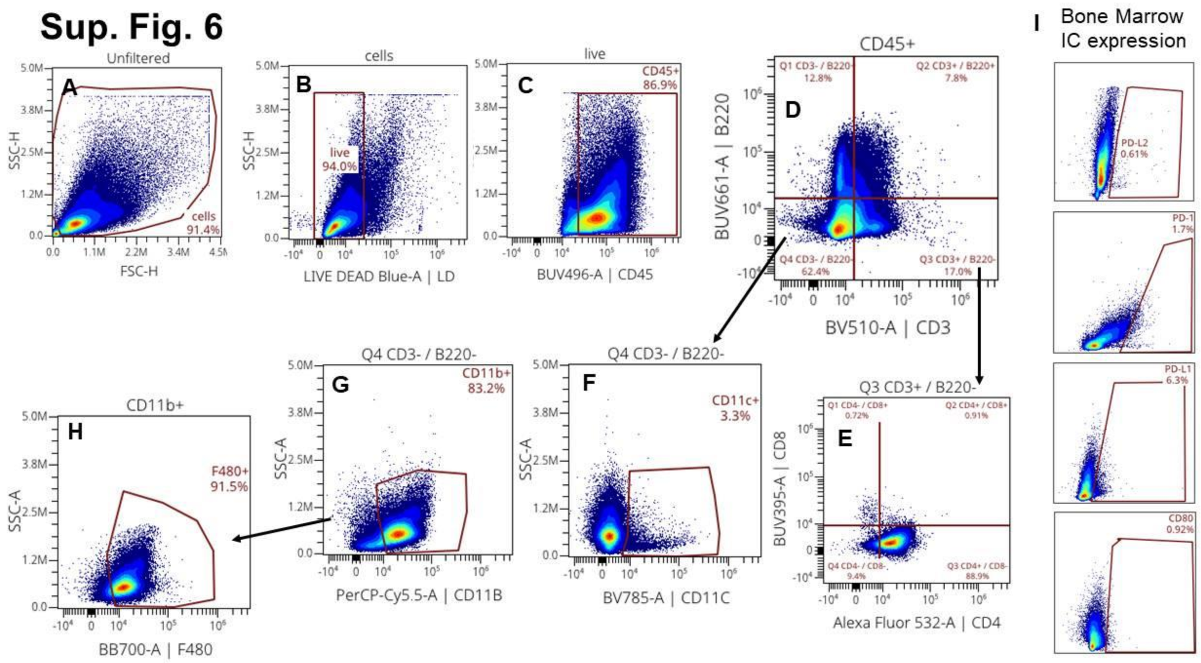

Sup. Fig. 6 Immune cell population gating scheme for bone marrow data from naïve mice

Gating schemes for immune cell populations depicted in Figs. 3. **(A)** Total cells **(B)** Live cells in total cell gate **(C)** CD45$^+$ cells in live cells **(D)** B220$^+$ and/or CD3$^+$ in CD45$^+$ B220$^+$CD3$^-$ were termed “B cells”, B220$^-$CD3$^+$ were termed “total T cells” **(E)** CD8$^+$ and/or CD4$^+$ in B220$^-$CD3$^+$ **(F)** CD11c$^+$ in B220$^-$CD3$^-$ were termed “myeloid DC” **(G)** CD11b$^+$ in B220$^-$CD3$^-$ were termed “total myeloid cells” **(H)** F4/80$^+$ in CD11b$^+$ were termed “macrophages” **(I)** PD-L2, PD-1, PD-L1 and CD80 immune checkpoint expression gating **(A-I)** OMIQ software was used to analyze and gate all data. N= 3-6 mice/group.

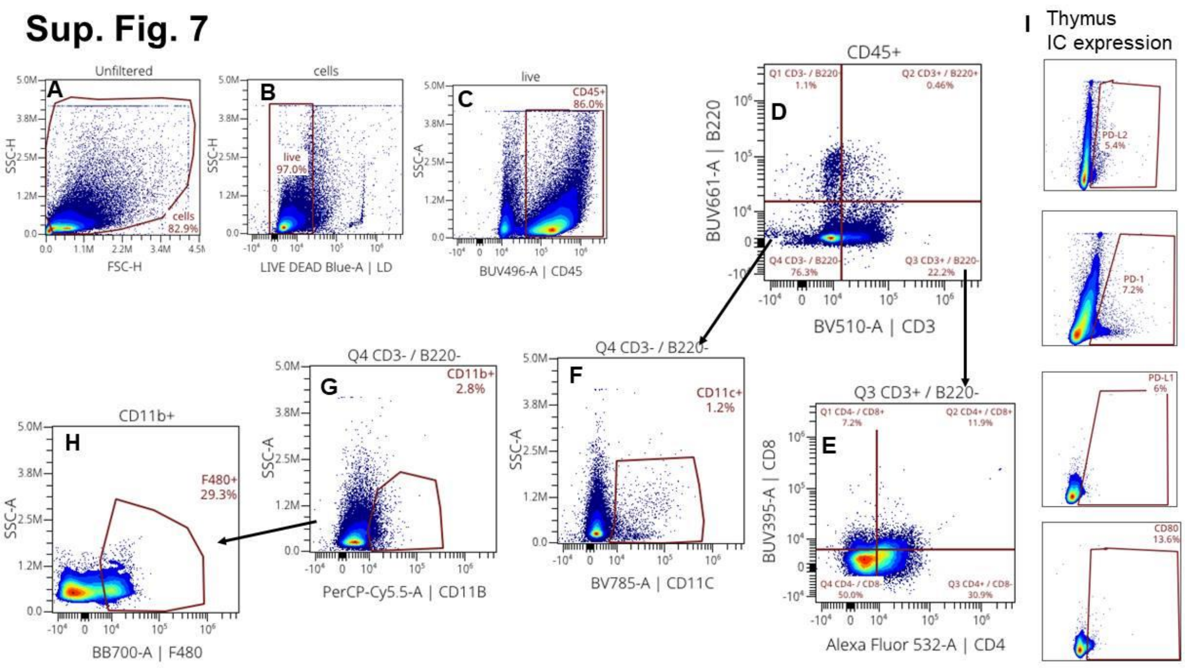

Sup. Fig. 7 Immune cell population gating scheme for thymus data from naïve mice

Gating schemes for immune cell populations depicted in Figs. 4-5. **(A)** Total cells **(B)** Live cells in total cell gate **(C)** CD45$^+$ cells in live cells **(D)** B220$^+$ and/or CD3$^+$ in CD45$^+$ B220$^+$CD3$^-$ were termed “B cells”, B220$^-$CD3$^+$ were termed “total T cells” **(E)** CD8$^+$ and/or CD4$^+$ in B220$^-$CD3$^+$ **(F)** CD11c$^+$ in B220$^-$CD3$^-$ were termed “myeloid DC” **(G)** CD11b$^+$ in B220$^-$CD3$^-$ were termed “total myeloid cells” **(H)** F4/80$^+$ in CD11b$^+$ was termed “macrophages” **(I)** PD-L2, PD-1, PD-L1 and CD80 immune checkpoint expression gating **(A-I)** OMIQ software was used to analyze and gate all data. N= 3-6 mice/group.

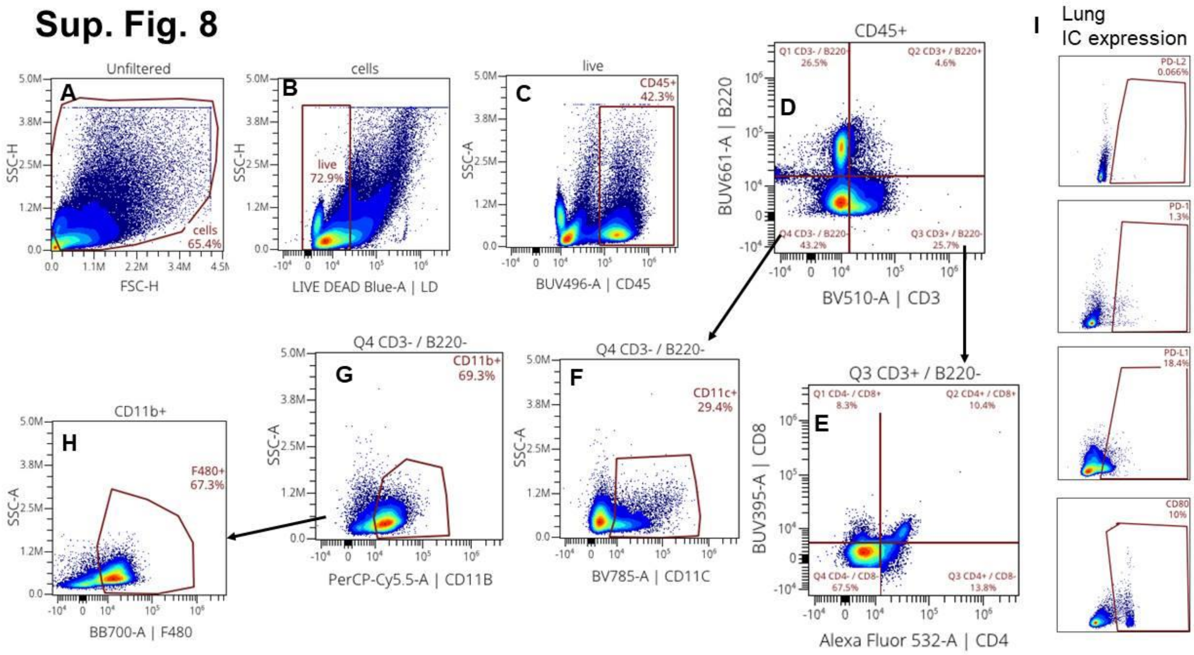

Sup. Fig. 8 Immune cell population gating scheme for lung data from naïve mice

Gating schemes for immune cell populations depicted in Figs. 4-5. **(A)** Total cells **(B)** Live cells in total cell gate **(C)** $CD45^+$ cells in live cells **(D)** $B220^+$ and/or $CD3^+$ in $CD45^+$ $B220^+CD3^-$ were termed “B cells”, $B220^-CD3^+$ were termed “total T cells” **(E)** $CD8^+$ and/or $CD4^+$ in $B220^-CD3^+$ **(F)** $CD11c^+$ in $B220^-CD3^-$ were termed “myeloid DC” **(G)** $CD11b^+$ in $B220^-CD3^-$ were termed “total myeloid cells” **(H)** $F4/80^+$ in $CD11b^+$ were termed “macrophages” **(I)** PD-L2, PD-1, PD-L1 and CD80 immune checkpoint expression gating **(A-I)** OMIQ software was used to analyze and gate all data. N= 3-6 mice/group.

Sup. Fig. 9

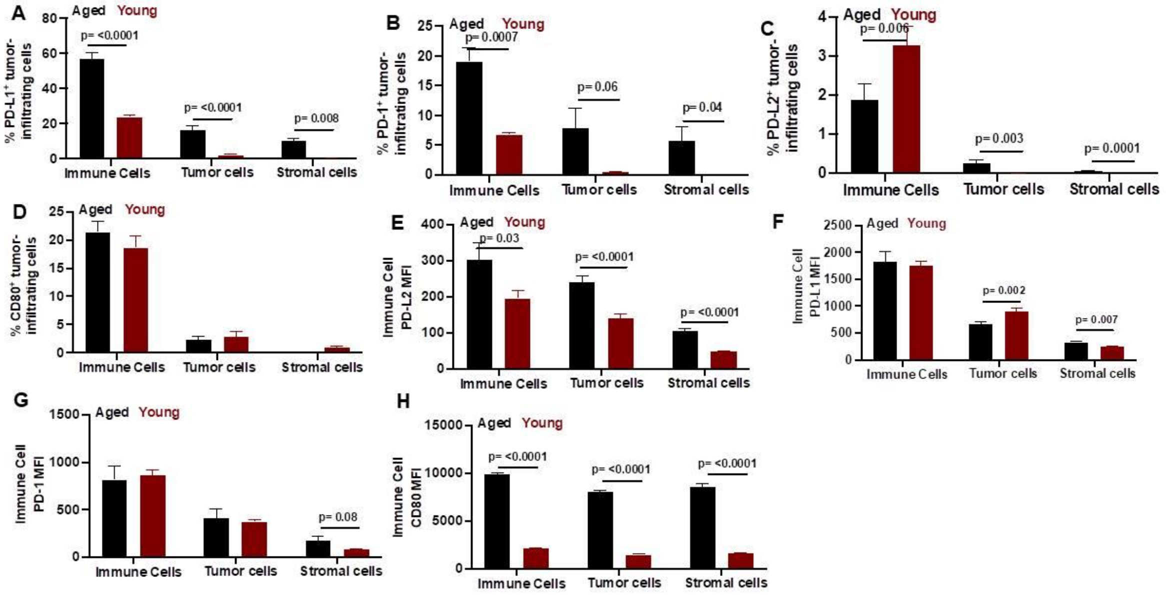

Sup. Fig. 9 Tumor-infiltrating cell CD80, PD-L1, PD-L2 and PD-1 expression in aged vs. young mice

Flow cytometry data on tumor-infiltrating immune cells and IC expression in aged (23-26 months) versus young (2-4 months) mice. (**A-H)** wild-type aged and wild-type young mice **(A)** PD-L1^{+} tumor infiltrating cells prevalence **(B)** PD-1^{+} tumor infiltrating cell prevalence **(C)** PD-L2^{+} tumor infiltrating cell prevalence, **(D)** CD80^{+} tumor infiltrating cell prevalence **(E)** PD-L2 MFI, **(F)** PD-L1 MFI, **(G)** PD-1 MFI, **(H)** CD80 MFI **(A-H)** Cell population gating is live CD45^{+} for immune cells, live CD45^{-}SSC^{hi} for tumor cells and live CD45^{-}SSC^{lo} for stromal cells. Standard error of mean indicated; *p*-values, students t-test. N= 6-9 tumors/group.

Sup. Fig. 10

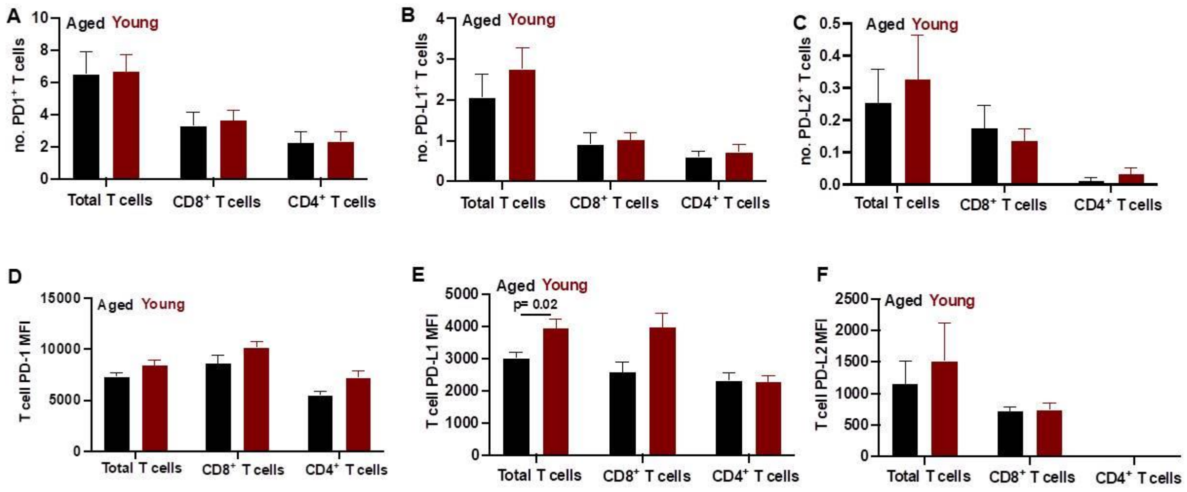

Sup. Fig. 10 Tumor-infiltrating T cell CD80, PD-L1, PD-L2 and PD-1 expression in aged versus young mice

Flow cytometry data on tumor-infiltrating immune T cells and IC expression prevalence in aged (23-26 months) versus young (2-4 months) mice. (**A-F)** wild-type aged and wild-type young mice **(A)** number PD-1$^+$ tumor infiltrating T cells **(B)** number PD-L1$^+$ tumor infiltrating T cells **(C)** number PD-L2$^+$ tumor infiltrating T cells , **(D)** PD-1 MFI **(E)** PD-L1 MFI, **(F)** PD-L2 MFI. **(A-F)** Population gating is CD45$^+$B220$^-$CD3$^+$ for total T cells of CD3$^+$ population and CD8$^+$ or CD4$^+$. Standard error of mean indicated; *p*-values, Student's *t*-test. N= 6-9 tumors/group.

Sup. Fig. 11

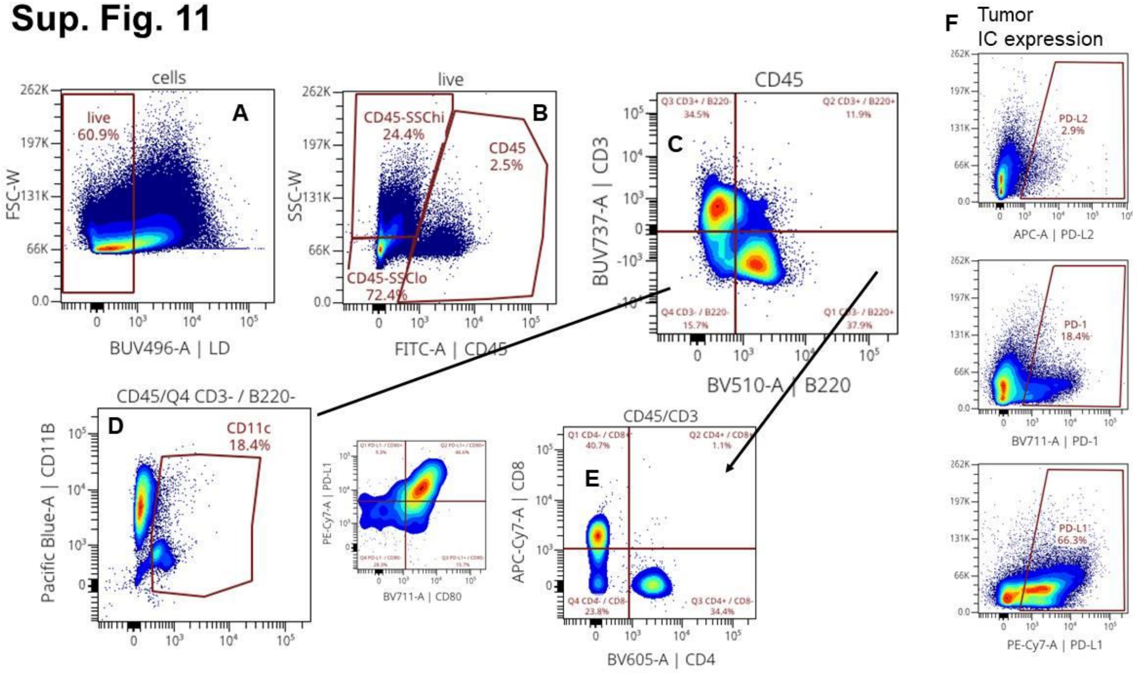

Sup. Fig. 11 Immune cell population gating scheme for tumor-infiltrating cells

Gating schemes for immune cell populations depicted in Figs. 6-8 **(A)** Live cells in total cells **(B)** $CD45^{+}$ termed "immune cells", $CD45^{-}$ SSC^{hi} termed "tumor cells", $CD45^{-}$ SSC^{lo} termed "stromal cells" in live cells **(C)** $B220^{+}$ and/or $CD3^{+}$ in $CD45^{+}$ $B220^{+}CD3^{-}$ were termed "B cells", $B220^{-}CD3^{+}$ were termed "total T cells" **(D)** $CD11c^{+}$ in $B220^{-}CD3^{-}$ was termed "myeloid DC" and PD-L1/CD80 co-expression on DC **(E)** $CD8^{+}$ and/or $CD4^{+}$ in $B220^{-}CD3^{+}$ **(F)** PD-L2, PD-1 and PD-L1 immune checkpoint expression gating. **(A-F)** OMIQ software was used to analyze and gate all data. N= 6-9 mice/group.

Sup. Fig. 12

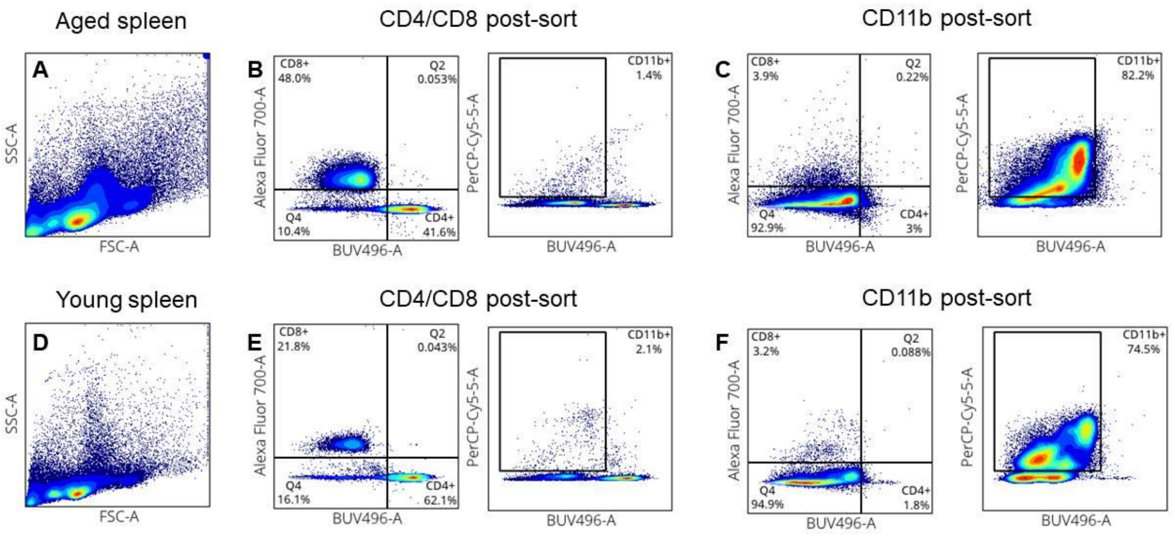

Sup. Fig. 12 post-sort populations used in co-culture experiments

Aged and young splenic post-sort populations. **(A)** Total cells in aged spleen **(B)** Post-sort populations in aged spleen sorted for $CD4^+$/$CD8^+$ T cells indicating CD4/CD8 expression and CD11b expression **(C)** Post-sort populations in aged spleen sorted for $CD11b^+$ myeloid cells indicating CD11b expression and CD4/CD8 expression **(D)** Total cells in young spleen **(E)** Post-sort populations in young spleen sorted for $CD4^+$/$CD8^+$ T cells indicating CD4/CD8 expression and CD11b expression **(F)** Post-sort populations in young spleen sorted for $CD11b^+$ myeloid cells indicating CD11b expression and CD4/CD8 expression. N=3-4 young or aged mice.
